# Multiple-low-dose therapy: effective killing of high-grade serous ovarian cancer cells with ATR and CHK1 inhibitors

**DOI:** 10.1093/narcan/zcac036

**Published:** 2022-11-12

**Authors:** Anya Golder, Louisa Nelson, Anthony Tighe, Bethany Barnes, Camilla Coulson-Gilmer, Robert D Morgan, Joanne C McGrail, Stephen S Taylor

**Affiliations:** Division of Cancer Sciences, School of Medical Sciences, Faculty of Biology, Medicine and Health, University of Manchester, and Manchester Cancer Research Centre, Wilmslow Road, Manchester M20 4GJ, UK; Division of Cancer Sciences, School of Medical Sciences, Faculty of Biology, Medicine and Health, University of Manchester, and Manchester Cancer Research Centre, Wilmslow Road, Manchester M20 4GJ, UK; Division of Cancer Sciences, School of Medical Sciences, Faculty of Biology, Medicine and Health, University of Manchester, and Manchester Cancer Research Centre, Wilmslow Road, Manchester M20 4GJ, UK; Division of Cancer Sciences, School of Medical Sciences, Faculty of Biology, Medicine and Health, University of Manchester, and Manchester Cancer Research Centre, Wilmslow Road, Manchester M20 4GJ, UK; Division of Cancer Sciences, School of Medical Sciences, Faculty of Biology, Medicine and Health, University of Manchester, and Manchester Cancer Research Centre, Wilmslow Road, Manchester M20 4GJ, UK; Division of Cancer Sciences, School of Medical Sciences, Faculty of Biology, Medicine and Health, University of Manchester, and Manchester Cancer Research Centre, Wilmslow Road, Manchester M20 4GJ, UK; Department of Medical Oncology, The Christie NHS Foundation Trust, Wilmslow Rd, Manchester M20 4BX, UK; Division of Cancer Sciences, School of Medical Sciences, Faculty of Biology, Medicine and Health, University of Manchester, and Manchester Cancer Research Centre, Wilmslow Road, Manchester M20 4GJ, UK; Division of Cancer Sciences, School of Medical Sciences, Faculty of Biology, Medicine and Health, University of Manchester, and Manchester Cancer Research Centre, Wilmslow Road, Manchester M20 4GJ, UK

## Abstract

High-grade serous ovarian cancer (HGSOC) is an aggressive disease that typically develops drug resistance, thus novel biomarker-driven strategies are required. Targeted therapy focuses on synthetic lethality—pioneered by PARP inhibition of *BRCA1/2*-mutant disease. Subsequently, targeting the DNA replication stress response (RSR) is of clinical interest. However, further mechanistic insight is required for biomarker discovery, requiring sensitive models that closely recapitulate HGSOC. We describe an optimized proliferation assay that we use to screen 16 patient-derived ovarian cancer models (OCMs) for response to RSR inhibitors (CHK1i, WEE1i, ATRi, PARGi). Despite genomic heterogeneity characteristic of HGSOC, measurement of OCM proliferation was reproducible and reflected intrinsic tumour-cell properties. Surprisingly, RSR targeting drugs were not interchangeable, as sensitivity to the four inhibitors was not correlated. Therefore, to overcome RSR redundancy, we screened the OCMs with all two-, three- and four-drug combinations in a multiple-low-dose strategy. We found that low-dose CHK1i-ATRi had a potent anti-proliferative effect on 15 of the 16 OCMs, and was synergistic with potential to minimise treatment resistance and toxicity. Low-dose ATRi-CHK1i induced replication catastrophe followed by mitotic exit and post-mitotic arrest or death. Therefore, this study demonstrates the potential of the living biobank of OCMs as a drug discovery platform for HGSOC.

## INTRODUCTION

Many established targeted cancer therapies directly inhibit the gene product of a recurrent, cancer-driving mutation. In such cases, drug development focuses on inhibitors that specifically target the driver, the presence of which is itself the biomarker for identifying the patient sub-populations most likely to benefit. Indeed, directly targeting *HER2* amplification, *EGFR* and *BRAF* mutations, or the *BCR-ABL* fusion kinase, transformed the treatment of breast and lung cancers, melanoma, and chronic myelogenous leukaemias, respectively (1–4). However, analogous actionable oncogenes are not frequent drivers in many other cancers, making this directly targeted approach more challenging where the disease is driven by loss-of-function mutations, for example in the case of tumour suppressor genes.

As an alternative, synthetic lethal strategies have emerged—pioneered by the finding that PARP inhibitors effectively target tumours lacking functional *BRCA1/2* (5,6). In this case, the development of selective inhibitors preceded biomarker discovery. Defining predictive biomarkers for synthetic lethal strategies can be challenging, highlighted by the difficulty in defining *BRCA1/2-*wildtype disease with homologous recombination deficiency that is sensitive PARP inhibition (7). Nonetheless, the success of PARP inhibitors led to the development of novel inhibitors targeting the DNA replication stress response (RSR), for which biomarkers related to RSR have been proposed (8). However, defining biomarkers for these novel agents has also not been straightforward, with added complexity from the potential role of biological processes more distal to the drug target, such as apoptotic responses and cell cycle control mechanisms (9). Therefore, work is required to develop unbiased strategies to aid pre-clinical biomarker discovery.

One approach to biomarker identification is to screen for synthetic lethal genes using a resistant cancer cell line and CRISPR/Cas9 or RNAi technology (10,11). However, this strategy is predicated on a single gene dictating sensitivity and will exclude non-genetic sources of synthetic lethality. An alternative unbiased approach is to identify sources of intrinsic synthetic lethality by directly comparing differentially sensitive cells, and deep phenotyping to determine the underling mechanisms to guide biomarker discovery. This approach requires a collection of *in vitro* models that closely recapitulate the cancer of interest, and a high-throughput assay to categorize models as sensitive or resistant to the drug(s) under evaluation. Pre-clinical studies and drug discovery efforts have largely relied on collections of established cell lines as models because they are tractable for mechanistic studies and drug-sensitivity profiling. However, cell lines often lack clinical annotation, and extensive *in vitro* culture results in loss of heterogeneity and genetic drift from the original tumour cells (12–14). Living biobanks have the potential to address many of these limitations; since cells are not extensively cultured *in vitro*, they closely reflect the molecular characteristics and heterogeneity of the original tumour (15–19). The potential for large collections of models in living biobanks presents the opportunity for extensive high-throughput screens to gain insight into the mechanism of sensitivity to novel inhibitors.

To facilitate mechanistic studies and biomarker development, we have established a biopsy pipeline, collecting samples from patients with ovarian cancer treated at The Christie Hospital, and a workflow to generate purified tumour fractions with extensive proliferative potential (15,20,21). This living biobank of ovarian cancer models (OCMs) has largely focused on high-grade serous ovarian cancer (HGSOC), an aggressive disease where treatment resistance is common meaning there is significant unmet need for novel biomarker-driven strategies (22). OCMs are clinically annotated and retain the molecular characteristics of the primary tumour, including p53 mutation and significant karyotype heterogeneity, reflecting ongoing chromosomal instability (15). OCMs can also be studied at early passage, before extensive genetic drift, and are amenable to mechanistic studies, as well as *omics* analyses. The OCMs therefore provide a platform to identify novel treatment strategies, and associated biomarkers, for patients with HGSOC. However, a key question is whether OCMs can serve as reliable models for drug sensitivity profiling.

We first set out to determine the potential of the OCMs for evaluating novel therapies by developing and optimizing a drug-sensitivity profiling assay based on proliferation. We opted to quantitate cell number directly during treatment using time-lapse microscopy, by visualizing chromatin labelled with GFP. Since the RSR is a known vulnerability of HGSOC (23,24), we then utilized this assay in a high-throughput screen using 16 OCMs to compare four different inhibitors of the RSR (ATR, CHK1, WEE1, PARG). Surprisingly our screen finds that sensitivity to these agents is largely not correlated, suggesting that a common RSR vulnerability is not driving sensitivity to the individual agents. We therefore screened all possible one-, two-, three- and four-drug combinations of these agents, as part of a multiple-low-dose (MLD) strategy (25–27). MLD is an attractive strategy for several reasons (25–27): (i) targeting multiple nodes of a single signalling pathway with a low doses can collectively result in complete pathway inhibition; (ii) use of low doses avoids the focused selective pressure that may result in treatment resistance; (iii) use of low doses may be less likely to result in unacceptable toxicity. Our MLD screen demonstrated that the vast majority of OCMs were sensitive to low-dose combinations of inhibitors targeting ATR and CHK1 or WEE1 and CHK1. Phenotypic analysis in turn shows that that low-dose ATR and CHK1 inhibition is highly synergistic when combined, inducing DNA replication catastrophe, leading to post-mitotic cell death, cell cycle arrest or senescence.

## MATERIALS AND METHODS

### *Ex vivo* ovarian cancer models

Research samples were obtained with informed patient consent from the Manchester Cancer Research Centre (MCRC) Biobank. The MCRC Biobank is licensed by the Human Tissue Authority (license number: 30004) and is ethically approved as a research tissue bank by the South Manchester Research Ethics Committee (Ref: 18/NW/0092). The role of the MCRC Biobank is to distribute samples and does not endorse studies performed or the interpretation of results. For more information, see https://www.mcrc.manchester.ac.uk/research/mcrc-biobank. Sixteen *ex vivo* OCMs were expanded from ascites samples from 14 patients, as published previously (15). Although all patients were initially diagnosed with HGSOC, four were re-classified upon histopathological review (Table 1) (20). Mean age at diagnosis was 62.5 years (range 25–84). Three samples were chemo-naïve (patients 38, 87 and 195).

**Table 1.** Patient and OCM characteristics

Patient	Anatomical site	Histology	FIGO stage	gBRCAm	Lines of CTx^†^	OCM	Seeding density (96-well)	References
33	PP	HGSOC	4B	Unknown	2	33–2	2000	Nelson *et al.*, Barnes *et al.*, Coulson-Gilmer *et al.*
38	OV/PP	HGSOC	3C	Unknown	0	38	2000	Nelson *et al.*, Barnes *et al.*
46	OV/PP	HGSOC	3C	Unknown	1	46–3	1000	Nelson *et al.*, Barnes *et al.*, Coulson-Gilmer *et al.*
59	OV/PP	HGSOC	3C	Unknown	2	59–3	4000	Nelson *et al.*, Barnes *et al.*, Coulson-Gilmer *et al.*
64	OV/PP	Possible HGSOC/LGSOC mix*	3C	Unknown	2	64–1	1000	Nelson *et al.*, Barnes *et al.*, Coulson-Gilmer *et al.*^§^
					3	64–3^-^	2000	
66	OV/PP	HGSOC	3C	Unknown	1	66–1	2000	Nelson *et al.*, Barnes *et al.*, Coulson-Gilmer *et al.*^§^
					1	66–5	3000	
72	OV	Moderately differentiated MUC	1A	Unknown	1	72	2000	Nelson *et al.*, Barnes *et al.*
79	OV	HGSOC	3C	Unknown	3	79	2000^‡^	Nelson *et al.*, Barnes *et al.*
87	OV	Possible CCOC*	3B	Unknown	0	87	1000	Nelson *et al.*, Barnes *et al.*, Coulson-Gilmer *et al.*
105	OV/PP	HGSOC	3C	*BRCA1 VUS*	3	105	1000	Coulson-Gilmer *et al.*
109	OV	HGSOC	4B	Unknown	3	109	5000	Barnes *et al.*, Coulson-Gilmer *et al.*
152	OV	Moderately differentiated serous adenocarcinoma of intermediate grade*	3C	Unknown	5	152	4000	Barnes *et al.*, Coulson-Gilmer *et al.*
191	OV	HGSOC	3A	*BRCA1*	2	191	4000	Barnes *et al.*, Coulson-Gilmer *et al.*
195	PP	Possible LGSOC*	4A	Unknown	0	195	4000	Barnes *et al.*, Coulson-Gilmer *et al.*

*See Barnes *et al.*, 2021; ^†^at time of research biopsy; ^‡^150000, 500, 2000, 4000 for 6-well, 12-well (colony formation assay), 24-well (time-lapse), coverslips, respectively; ^§^Coulson-Gilmer *et al.*, only included 64-1 and 66-1 (and not subsequent OCMs from these patients). CCOC, clear cell ovarian cancer; CTx=chemotherapy; gBRCAm=patient germline *BRCA1/2* mutation; HGSOC=high-grade serous ovarian cancer; LGSOC=low-grade serous ovarian cancer; MOC=mucinous ovarian cancer; OV=ovary; PP=primary peritoneum; VUS, variant of uncertain clinical significance.

Note that the culture established from the third biopsy from patient 64 was further separated into EpCAM-negative (64-3-) and EpCAM-positive cells, with only OCM.64-3- included in the current analysis. See also Supplementary Figure S1.

Analysis by exome sequencing for 11 of the OCMs has been reported and data deposited previously (15). For analysis of the panel of 11 genes in the present study, reads were filtered and aligned to the human reference sequence analysis set (hg38/Dec. 2013/GRCh38) from the UCSC browser (28), using BWA-MEM v0.7.1566 (29). Single nucleotide variants, indels and copy number variants (including in matched tumour and stromal cells) were called using Varscan 2 (v.2.4.0) (30), using samtools mpileup as input. Variants (SNVs and indels) were classified as: germline if tumour and stromal sequence match but are different to reference genome; somatic if tumour and stromal sequence do not match (but stromal sequences matches reference) and there is a significant difference in allele frequency; or loss of heterozygosity if the stromal sequence is heterozygous but the tumour sequence is homozygous. Functional annotation of genetic variants was performed using ANNOVAR (v20191024) (31) with the GENCODE (v40) Basic set (32). Pathogenicity of germline variants was determined by cross-referencing with ClinVar (33). However, no germline variants identified in the 11 OCMs were annotated as ‘pathogenic’ or ‘likely pathogenic.’ Somatic variants were filtered to those giving rise to changes in the protein sequence i.e. exonic, splice variants and truncating mutations, or those in regulatory sequences. Varscan 2 identified copy number changes were further processed as recommended in the user guide. Raw copy number calls were adjusted for GC content and preliminary calls made using the copyCaller function of Varscan2. Copy number data was then segmented using a circular binary segmentation algorithm in the DNAcopy (v.1.70.0) (34) library from Bioconductor (v.3.15) in R (v.4.1.0). Adjacent segments of similar copy number were merged using an accessory script provided with Varscan 2. These segment coordinates were then aligned with the GENCODE (v40) Basic set to give copy number state for every gene. SNVs, indels and copy number state for genes of interest was visualised by oncoprint (35,36).

Analysis by RNA-sequencing has been reported previously for the 16 OCMs (15,20,21). For the analysis presented here, read counts were processed using the deseq2 (37) package from Bioconductor in R and a variance stabilising transformation was performed. The R package ComplexHeatmap (38) was then used to visualise gene expression profiles.

### Cell culture

OCMs were cultured in OCMI media (39) as described previously (15). FNE1 cells (a kind gift from Tan A. Ince) were cultured in WIT-Fo Culture Media FOMI media as described previously (40). For long-term storage, cells were frozen in Bambanker (Wako pure chemical) and stored in a LN_2_ storage vessel. Cells were periodically tested for the presence of mycoplasma by the Molecular Biology Core Facility at the CRUK Manchester Institute. PDD00017273 (PARGi; Tocris), AZD6738 (ATRi; AstraZeneca), AZD7762 (CHK1i; AstraZeneca), AZD1775 (WEE1i; Salleck Chem), EPT46464 (Salleck Chem) and LY2603618 (Salleck Chem) were all dissolved in DMSO and stored below -20°C and used as described in figure legends. Hydroxyurea (Sigma Aldrich) and gemcitabine (Salleck Chem) were dissolved in water and used as described in figure legends.

### Lentiviral transduction

AAV293T cells (Agilent Technologies) were transfected with either pLVX-myc-EmGFP-H2B, or pLVX-myc-H2B-mCherry, along with psPAX2 and pMD2.G (gifts from Didier Trono via Addgene) using CaCl_2_ (Promega) in DMEM supplemented with 10% Hyclone serum (GE Healthcare) and incubated overnight. Virus was harvested 48 later, centrifuged, filtered then added to OCMs with 10 μg/ml polybrene (Sigma Aldrich) and the cells centrifuged at 300 × g, 30 °C for 2.5 h followed by overnight incubation. Puromycin (Sigma Aldrich) (1 μg/ml) was added 48 h after transduction.

### Cell fate profiling

Cells were seeded in black μclear^®^ 96-well plates (Greiner Bio-One) 24 h prior to drug addition. Plates were imaged using an IncuCyte^®^ ZOOM (Satorius AG) using an 20× objective and maintained at 37°C in a humidified 5% CO_2_ and 5% O_2_ atmosphere. Phase images were acquired at 10-min intervals over 96 h and imaging sequences were exported in MPEG-4 format and analyzed manually.

### Time-lapse microscopy drug profiling assay

Cells expressing fluourescent-H2B were seeded in black, μclear^®^ 96-well plates (Greiner Bio-One), at densities given in Table 1, with drugs added 24 h post seeding. Plates were imaged immediately using an IncuCyte^®^ ZOOM (Satorius AG), maintained at 37°C in a humidified 5% CO_2_ and 5% O_2_ atmosphere, with images acquired once every 1–6 h for a maximum of 144 h. IncuCyte^®^ ZOOM software was used in real-time to measure fluorescent object count. Fluorescent object count was normalised to *t* = 0 for each drug concentration to generate a proliferation curve. The area under the proliferation curve (AUC) was calculated for each drug concentration to generate a dose-response curve with non-linear regression used to determine GI values. To determine the doubling time for each culture, the following equation was used:

$$\begin{equation*}N\ \left( t \right) = {N}_0\ {2}^{t/{T}_d}\end{equation*}$$

where *N*(*t*) = the number of objects at time *t*, *T*_d_ = doubling period (time it takes for object to double in number), *N*_0_ = initial number of objects and *t* = time. A log_2_ transformation was performed on the normalized fluorescent count of untreated cells. The data were plotted against time and the inverse gradient of the log-phase portion of the graph calculated to give culture doubling time.

For the first stage of the MLD drug combination screen, the time-lapse microscopy drug profiling assay was used to measure the 10% maximal growth inhibition concentration (GI_10_) of PDD00017273 (PARGi), AZD6738 (ATRi), AZD7762 (CHK1i) and AZD1775 (WEE1i) for each OCM. For the second stage, the GI_10_ doses from the four drugs were combined in double-, triple- and four-drug combinations for each OCM. For OCMs where a GI_10_ could not be determined, a generic low dose was used (i.e. PARGi = 100 nM, ATRi = 2 μM). As detailed above, the time-lapse microscopy drug profiling assay was used to image cells for 96 h and the AUC of the normalized fluorescent count determined. The proliferation rate for each drug combination was calculated by normalization of the AUC to untreated cells. Due to batch-to-batch variation between commercial drug supplies and stability when reconstituted, GI_10_ values for mechanistic analyses were re-calculated periodically.

### Cell viability drug profiling assay

Cells were seeded in black μclear^®^ 96-well plates (Greiner Bio-One) with the addition of drugs 24 h later. After a 72 h incubation with drugs, media was replaced with a 1:1 ratio of media and CellTiter-Glo^®^ 2.0 reagent (Promega). Luminescent signal was measured using a Varioskan™ LUX multimode microplate reader (Thermo Scientific), with readings normalised to blank wells containing reagent only.

### Colony formation assay

Cells were seeded into 12-well plates at 500 cells/well before addition of drugs 24 h later. Media containing drugs was replaced at every 3–4 days, with fixation of cells in 1% (v/v) formaldehyde (Fisher Scientific) occurring when either one well on the plate reached confluence, or after 4 weeks treatment. Cells were stained with 0.05% (w/v) crystal violet solution (Sigma-Aldrich), before imaging using a ChemiDoc™ Touch Imaging System (Bio-Rad). Quantification of the colony area was performed using ImageJ software (NIH) with the ColonyArea plugin.

### Immunoblotting

Protein was extracted by boiling cell pellets in SDS buffer (0.35 M Tris pH 6.8, 0.1 g/ml sodium dodecyl sulphate, 93 mg/ml dithiothreitol, 30% (v/v) glycerol, 50 μg/ml bromophenol blue; all from Sigma Aldrich) and resolved by SDS-PAGE using NuPAGE™ 4–12% (v/v) Bis-Tris protein gels (1.0 mm) (Life Technologies), before electroblotting onto methanol-soaked Immobilon-P nitrocellulose membranes (Merck Millipore). Membranes were blocked in either 5% (w/v) dried skimmed milk (Marvel), or for phospho-specific antibodies, 5% (w/v) bovine serum albumin (BSA, Sigma Aldrich) dissolved in TBST (50 mM Tris pH 7.6, 150 mM NaCl, 0.1% Tween-20), with membranes incubated overnight at 4°C with the following primary antibodies: mouse anti-CDC25A (Santa Cruz Biotechnology cat#sc-7389; RRID:AB_62722; 1:200); mouse anti-CHK1 (Santa Cruz Biotechnology, cat#sc-8408, RRID: AB_627657, 1:400); rabbit anti-phospho-CHK1 serine-296 (Cell Signalling, cat#2349, RRID:AB_2080323, 1:500); rabbit anti-phospho-CHK1 serine-345 (Cell Signalling, cat#2348, RRID:AB_331212, 1:750); sheep anti-Tao1 ((41), 1:1000). Membranes were washed 3 x TBS-T before incubating with the appropriate horseradish-peroxide (HRP)-conjugated secondary antibodies for a minimum of 2 hours (goat anti-mouse IgG [H + L] HRP, Invitrogen, cat#G21040 RRID: AB_2536527; goat anti-rabbit IgG [H+L] HRP, Invitrogen, cat#G21234, RRID: AB_ 1500696; rabbit anti-sheep IgG [H+L] HRP, Invitrogen, cat#618620, RRID: AB_2533942; all 1:2000). Following 3× TBS-T washes, bound secondary antibodies were detected using either EZ-Chemiluminescence Reagent (Geneflow Ltd) or Luminata™ Forte Western HRP Substrate (Merck Millipore) and a ChemiDoc™ Touch Imaging System (BioRad). Image Lab software (BioRad) and Adobe Photoshop^®^ CC 2018 (Adobe Systems Inc.) were used to process images.

### Immunofluorescence

Cells were seeded 24 h prior to drug treatment onto pre-coated 19 mm coverslips or 96-well Cell Carrier plates (PerkinElmer). Cells were fixed with addition of 1% (v/v) formaldehyde for 5 min, washed in PBS, before quenching in glycine (12.5 mM in PBS) for 5 min. Subsequent wash steps used PBS-T (PBS, 0.1% (v/v) Triton X-100). The following primary antibodies were added to cells for 30 min at room temperature: rabbit anti-Vimentin (EPR3776) (Abcam, cat#ab92547, RRID: AB_10562134, 1:1000); mouse anti-γH2AX pS139 (Merck Millipore, cat#05-636, RRID: AB_309864, 1:500); rabbit anti-RPA70 (Abcam, cat#ab79397, RRID: AB_1603759, 1:350). Cells were washed and incubated with fluorescently conjugated secondary antibodies for 30 min: donkey anti-mouse Cy3 (Jackson ImmunoResearch Laboratories Inc, cat#715–165-150, RRID: AB_2340813, 1:500), donkey anti-rabbit Cy5 (Jackson ImmunoResearch Laboratories Inc, cat#711–175-152, RRID: AB_2340607, 1:500). Cells were washed and DNA stained with 1 μg/ml Hoechst 33258 for 2 min at room temperature. Coverslips were mounted onto microscope slides (90% (v/v) glycerol, 20 mM Tris, pH 9.2) and stored at −20°C before image acquisition. For high-throughput immunofluorescence, 96-well plates were stored in PBS at 4°C prior to image acquisition.

For coverslips, image acquisition was performed using either a 40× or 63× objective on an Axioskop2 (Ziess, Inc.) microscope fitted with a CoolSNAP HQ camera (Photometrics). MetaMorph Software (Molecular Devices) with Adobe Photoshop^®^ CC 2018 (Adobe Systems Inc.) used for image processing. For high-throughput immunofluorescence, image acquisition was performed using Operetta^®^ High Content Imaging System (Perkin Elmer) with image analysis and quantitation using Harmony and Columbus High Content Imaging and Analysis Software (Perkin Elmer). Analysis software used Hoechst 33258 staining to calculate intensity thresholds and generate a nuclear mask. Pixel intensity of the relevant fluorescent antibody could be quantitated from within the nuclear area and used to calculate mean intensity or number of objects. For quantitation, a minimum of 1000 cells per condition were analyzed.

### DNA fibre assay

Asynchronous cells were incubated with inhibitors for 1 h, before double labelling with nucleoside analogues. Media containing 5 μM BrdU plus drug(s) was added for 20 min at 37°C, followed by 3× PBS washes, and addition of media containing 200 μM IdU plus drug(s) for 20 min at 37°C, followed by 2 wash steps using ice-cold PBS. Note, only the IdU track was analyzed for Figure 5. Cells were then trypsinised and resuspended in ice-cold PBS at 8×10^5^ cells/ml. For DNA spreading, 2 μl of the cell suspension was added to a SuperFrost Plus™ adhesion slide (Thermo Scientific) and allowed to dry for 5–10 min. 7 μl of spreading/lysis buffer (200 mM Tris–HCl pH 7.5, 50 mM EDTA, 0.5% (w/v) SDS) was added to the cell suspension, gently mixed and incubated for 2–3 min at room temperature. Slides were then tilted at a 3–10° angle to allow the suspension to slowly run down the slide and spread for several minutes. The spreads were then allowed to dry before fixation in methanol/acetic acid (3:1) for 10 min. Once dry, slides were stored at 4°C.

Before immunolabeling, slides were washed 2× ddH_2_O for 5 min and 1×2.5 M HCl, and denatured in 2.5 M HCl for 1h. Slides were then washed twice with PBS, 2× blocking solution (PBS, 1% (w/v) BSA, 0.1% (v/v) Tween20) and incubated in blocking solution for 1 h. For immunolabeling, all antibodies were diluted in blocking solution. To detect BrdU, slides were incubated with rat anti-BrdU primary antibody (Abcam, BU1/75 [ICR], cat#6326, RRID: AB_305426, 1:500) at room temperature for 1h, and washed 3 x PBS. Slides were fixed for 10 min in 1% (v/v) formaldehyde, washed 3× PBS and subsequently quenched in glycine (12.5 mM in PBS). Slides were washed 3× PBS, 3× blocking solution for 5 min, before addition of mouse anti-BrdU primary antibody (BD Biosciences, BU44, cat#347580, RRID: AB_400326, 1:100) to detect IdU overnight at 4°C. Post-incubation, cells were washed 2× PBS, 3× blocking solution for 5 min, before incubation with the following fluorescently-conjugated secondary antibodies for 2 h: donkey anti-mouse Cy2 (Jackson ImmunoResearch Laboratories Inc., cat#715-225-150, RRID: AB_2340826, 1:500); donkey anti-rat Cy3 (Jackson ImmunoResearch Laboratories Inc, cat#712-165-153, RRID: AB_2340667, 1:500). Final wash steps consist of 2× PBS, 3× blocking solution for 5 min and 2× PBS, before mounting slides to coverslips using PBS:Glycerol (1:1).

Image acquisition was with a 100× oil immersion objective on an Axioskop2 (Zeiss) microscope fitted with a CoolSNAP HQ camera (Photometrics). Fibre lengths were measured using ImageJ software (NIH) with a minimum of 300 IdU fibres measured.

### High-resolution fluorescence time-lapse microscopy

Cells expressing GFP-H2B were seeded in black μ-Plate 24-well plates (Ibidi) 24 h prior to treatment. Image acquisition occurred over 72 h using an Axiovert 200 inverted microscope (Zeiss) equipped with a ×40 Plan NEOFLUAR objective, an PZ-2000 automated stage (Applied Scientific Instrumentation) and an environmental control chamber (Solent Scientific), which maintained cells at 37°C in a humidified 5% CO_2_ atmosphere. MetaMorph software (Molecular Devices) was used to control shutters, filter wheels, and point visiting and an Evolve^®^ Delta camera (Photometrics) was used for image capture. Image sequences were analyzed manually using MetaMorph software and used to identify mitotic events.

### Synergy analysis

Cells were seeded 24 h before addition of drugs, which were titrated to form a drug concentration matrix. Proliferation was measured by either time lapse microscopy drug profiling assay or by cell viability assay at the 72-h time-point, with proliferation normalised to untreated cells. Combenefit software (42) was used to calculate pharmacologic interaction using the Loewe additivity model. Synergy matrices displayed the Loewe synergy value for each combination of drug concentrations. The ‘drug average synergy’ was also calculated, which is the concentration of one drug where synergy appears to be localized.

### Quantification and statistical analysis

Statistical analyses were performed using Prism 8 (GraphPad) to calculate doubling time, AUC, Pearson's r and GI values. In statistical analysis, ∗ *P* < 0.05, ∗∗ *P* < 0.01, ∗∗∗ *P* < 0.001, ∗∗∗∗ *P* < 0.0001, ns: *P* > 0.05. For bar graphs where sample means are used based on population measurements from a minimum of three experiments, the parametric one-way ANOVA statistical test is used as indicated in the figure legend. Error bars are described in the figure legends. For correlation analysis, a two-tailed *P* value was determined.

## RESULTS

### A time-lapse imaging workflow to determine OCM proliferation rates

**Figure 1.**
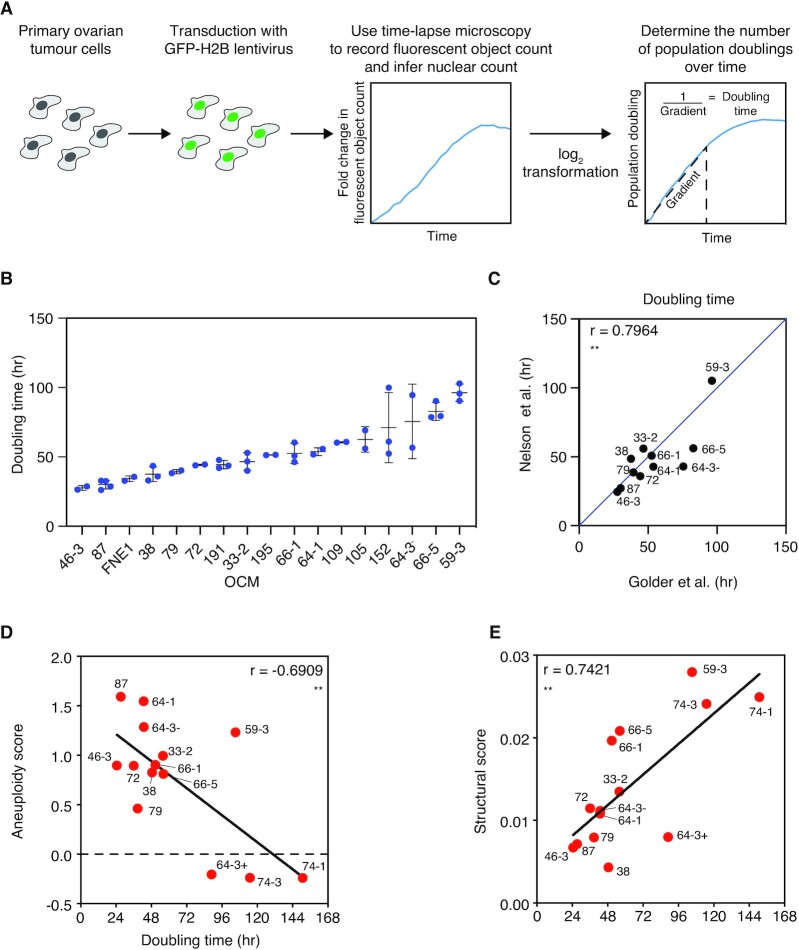
Measurement of OCM proliferation rates. (**A**) OCMs were transduced with a lentivirus expressing a GFP-tagged histone 2B (GFP-H2B) to generate cells with fluorescent nuclei. Time-lapse microscopy was used to measure fluorescent object count to infer nuclear count, as a proxy for cell number, over 120 h. The inverse gradient of the linear portion of a log_2_ transformation of the fluorescent object count, normalised to *t* = 0, was used to determine culture doubling time. (**B**) Doubling times of the present cohort of 16 OCMs and the non-transformed FNE1 cell line ranked from shortest to longest duration. Mean and SD determined from a minimum of two independent experiments. (**C**) Comparison of mean doubling time from (**B**) with Nelson *et al.*, 2020 for the 11 OCMs included in both analyses (note, for drug screening purposes the present cohort excludes OCMs with relatively slow proliferation rates and includes novel OCMs not available at the time of the prior analysis). A *y* = *x* line is shown for illustrative purposes. (**D**, **E**) Comparison of doubling time with aneuploidy score (D) and structural score (E) from shallow, single-cell, whole-genome sequencing (both datasets from Nelson *et al.*, 2020). Linear regression is shown and Pearson's *r* is used to measure correlation. Two-tailed *P* value, ** *P* ≤ 0.01. Note that the culture established from the third biopsy from patient 64 was further separated into EpCAM-negative (64-3-) and EpCAM-positive (64-3+) cells, with only OCM.64-3- included in the current analysis (15). See also Supplementary Figure S1.

We have built a living biobank to evaluate novel therapies for HGSOC. Starting with ascites, our workflow yields purified tumour fractions with extensive proliferative potential that can be maintained in standard 2D cell culture conditions (15). To determine whether these OCMs provide a tractable drug discovery platform, we set out to develop a high-throughput drug-sensitivity profiling assay. We elected to measure proliferation by labelling OCMs with a GFP-tagged histone to allow visualisation of the chromatin, followed by time-lapse microscopy to quantitate fluorescent objects as a proxy for nuclear count (Figure 1A). Doubling time was then calculated as the inverse gradient of the linear portion of a log_2_ transformation of the fluorescent object count, normalised to *t* = 0. To evaluate the reproducibility of this approach we analysed a cohort of 16 OCMs, many of which had been analysed previously (Table 1; Supplementary Figure S1) (15). As demonstrated previously, the culture dynamics of the cohort were highly heterogeneous, with doubling times ranging from 28 h for OCM.46–3 to 96 h for OCM.59–3 (Figure 1B). Importantly, doubling times remained largely consistent with our previous analysis generated over 18 months earlier (Figure 1C).

Because HGSOC have extensive CIN, we reasoned that the extremely long doubling times of some OCMs may reflect poor fitness due to highly abnormal genomes. To test this, we took advantage of available shallow single-cell, whole-genome sequencing data, and asked whether proliferation rate correlated with metrics of genomic abnormalities. We analysed aneuploidy scores, which measure divergence from euploidy, and structural aberration scores, which measure copy number state transitions (15,43). There was a significant negative correlation between OCM doubling time and aneuploidy score (*r* = −0.6909; Figure 1D), suggesting that deviation from the euploid state confers a proliferative advantage, a notion that runs counter to the prevailing view (44–47). However, OCMs 64-3^+^, 74-1 and 74-3, which exhibit very long doubling times, have negative aneuploidy scores because they possess numerous monosomies (15). This suggests that the correlation may be dominated by OCMs where chromosome losses have a negative impact on proliferation. Conversely, the structural aberration score positively correlated with doubling time (*r* = 0.7421), i.e. OCMs with high levels of structural rearrangements tend to proliferate more slowly (Figure 1E). OCM.59-3, for example, has the highest structural score and doubling time of 105 h. This correlation is more intuitive and consistent with the notion that extensive genomic abnormalities impart a fitness cost. Moreover, it suggests that the variable proliferation rates reflect intrinsic properties of the tumour cells. Finally, and most importantly, these observations show that the measurement of OCM proliferation rates using time-lapse tracking of nuclei is sufficiently reproducible, thus providing a suitable assay for drug sensitivity profiling.

### Drug-sensitivity profiling using a proliferation assay

**Figure 2.**
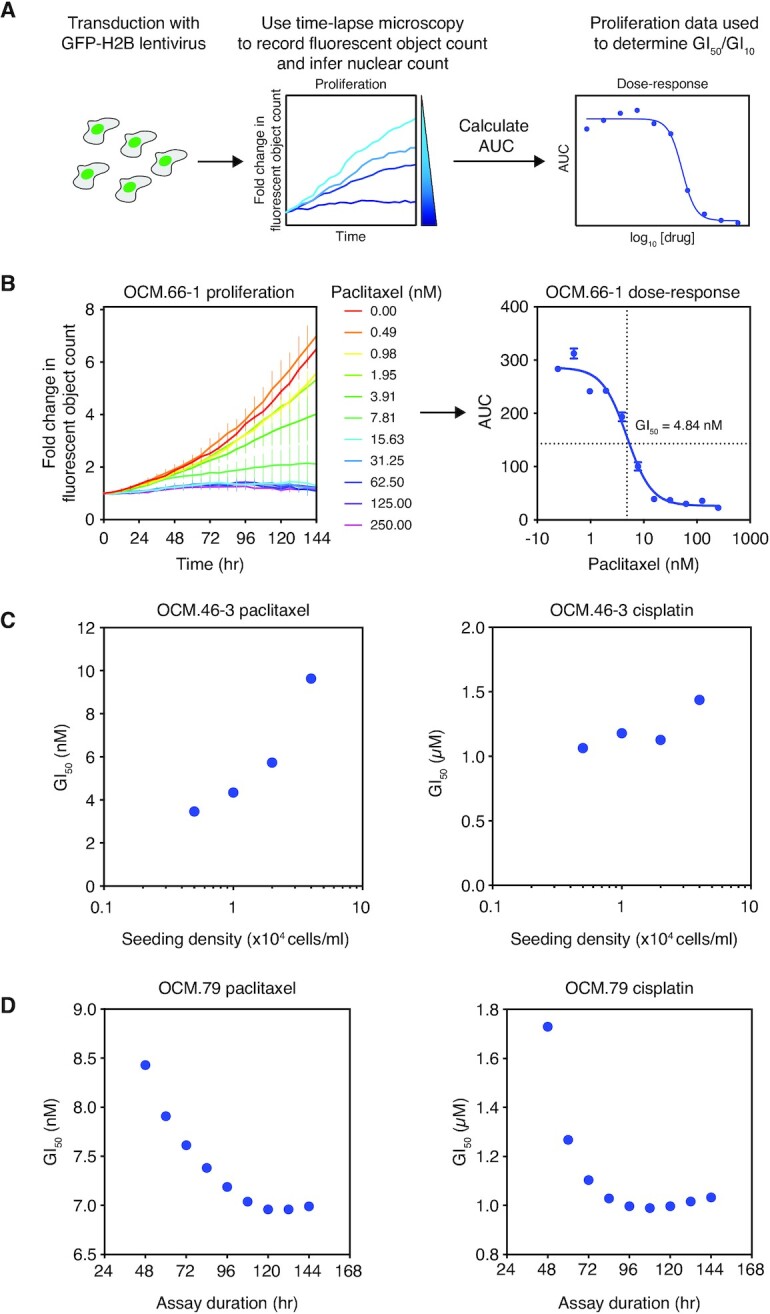
Drug sensitivity profiling of OCMs by time-lapse microscopy. (**A**) Schematic of the drug profiling assay. OCMs expressing GFP-H2B are treated with a drug titration and time-lapse microscopy used to plot fluorescent object count over time, normalised to *t* = 0. The area-under-the-curve (AUC) for each drug concentration is calculated and plotted onto a dose–response curve and GI_50_ determined. (**B**) Exemplar assay showing determination of the paclitaxel GI_50_ for OCM.66-1. Cells were treated with a titration of 0–250 nM paclitaxel with fluorescent object count measured over 96h and normalised to *t* = 0 (left panel). Each point represents the mean of three technical replicates ± SD. The corresponding dose-response curve plots the mean AUC ± SEM (right panel). (**C**) Paclitaxel (left panel) and cisplatin (right panel) GI_50_ of OCM.46-3 at various assay seeding densities. (**D**) Paclitaxel (left panel) and cisplatin (right panel) GI_50_ of OCM.79 at various assay durations. See also Supplementary Figure S2.

Having confirmed that measurement of proliferation using time-lapse microscopy is reproducible and reflective of intrinsic tumour cell characteristics, we extended this methodology into a short-term drug-sensitivity profiling assay. We first measured the half maximal growth inhibition concentration of drug (GI_50_) to reflect the drug sensitivity of the OCMs. GI values were determined using dose-response curves, generated by measuring the area-under-the-curve of fluorescent object count over time for a range of drug concentrations (Figure 2A, B). Quoted GI values are based on either single technical replicates or the mean of three technical replicates depending on the purpose of the experiment—see figure legends for details.

Our rationale for using fluorescent object count to measure drug sensitivity was based on experience analysing an inhibitor of the PAR glycohydrolase, PARG (PARGi; PDD00017273) (21,48). While many drug screens infer cell viability based on ATP metabolism, in cases where drugs have cytostatic effects viability assays can underestimate drug effects compared with direct cell counting (49). We confirmed this by comparing imaging- and metabolism-based assays to analyse the sensitivity of OCM.109 to PARGi. Treatment of PARGi-sensitive OCM.109 resulted in fewer cells but with rounded cytoplasms and enlarged nuclei (Supplementary Figure S2A, B), reminiscent of the ‘‘fried egg’’ morphology described previously (48). Overall, the time-lapse assay captured a reduction in proliferation of OCM.109 with PARGi treatment (Supplementary Figure S2C). Conversely, the presence of enlarged cells resulted in an underestimation of the decline in cell number by the metabolism-based assay (Supplementary Figure S2C). Thus, direct measurement of proliferation is the optimal approach for evaluating novel drugs of unknown mechanism, as it can discriminate between cell growth versus proliferation.

### Optimization of the proliferation assay for OCM drug-sensitivity profiling

Having established that an imaging-based proliferation assay was superior to a metabolism assay, we optimised a number of assay parameters. Cell seeding density and assay duration can affect drug-sensitivity profiling when there is variability in cell cycle duration (50–53). Since the OCMs exhibit significant heterogeneity in culture dynamics (Figure 1B), we optimised assay seeding density and assay duration. For this we used paclitaxel and cisplatin as they have different mechanisms of action and are used for the treatment of ovarian cancer (54). The GI_50_ of OCM.46-3 to both drugs increased as seeding density was increased but was more consistent at lower seeding densities (Figure 2C). Indeed, proliferation of untreated cells plateaued earlier as seeding density increased (Supplementary Figure S2D). Therefore, higher seeding densities likely give insufficient time for treatment to exert an anti-proliferative effect and artificially elevate GI_50_. Consequently, we identified the optimal seeding density for each of the 16 OCMs to allow a minimum of 72 hours proliferation (Table 1).

Conversely, as assay duration was increased, the GI_50_ of OCM.79 in response to both paclitaxel and cisplatin decreased (Figure 2D, Supplementary Figure S2E). For example, an increase in cisplatin treatment duration from 48 h (GI_50_ = 1.7 μM) to 120 h (GI_50_ = 1.0 μM) resulted in a 1.7-fold reduction in GI_50_. Since GI_50_ for both drugs decreased until a treatment duration of ∼96–108 h before stabilising, a minimum of 96 h was used for subsequent assays.

### Profiling the sensitivity of OCMs to inhibitors of the replication stress response

**Figure 3.**
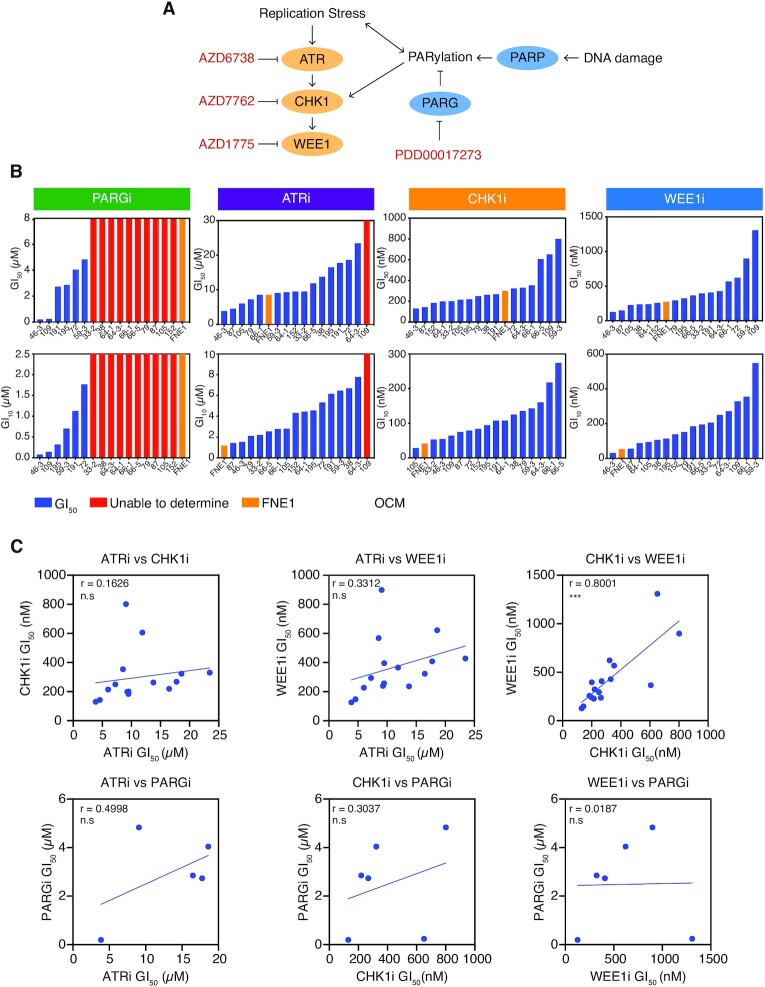
Drug-sensitivity profiling of OCMs with inhibitors of the replication stress response. (**A**) Inhibitors of the RSR used in the OCM drug-sensitivity screen, specifically targeting the replication checkpoint kinases and PARylation. Replication stress activates the ATR-CHK1-WEE1 signalling cascade (93). Replication stress and DNA damage impact PAR dynamics controlled by PARP and PARG, and PAR chains can also activate CHK1 directly (94,95). (**B**) Time-lapse microscopy drug-sensitivity profiling was used to determine the GI_50_ (upper panels) and GI_10_ (lower panels) of the 16 OCMs and FNE1 cells to PARGi, ATRi, CHK1i and WEE1i. Cells were treated with a dose-titration of each drug adapted for individual OCMs to allow optimal measurement of GI_50_ and GI_10_. Data from a single experiment. (**C**) Pairwise comparisons of OCM GI_50_ for PARGi, ATRi, CHK1i and WEE1i. Pearson's r is used to measure correlation. Two-tailed *P* value, *** *P* ≤ 0.001, n.s. *P* > 0.05. OCMs for which a GI_50_ was not determined in (B) were excluded from analysis. See also Supplementary Figure S3.

Upon optimisation of the drug-sensitivity profiling assay, we explored a number of inhibitors targeting the RSR, a known vulnerability in HGSOC (23,24). We screened the 16 OCMs for sensitivity to inhibitors of ATR (ATRi; AZD6738) (55), CHK1 (CHK1i; AZD7762) (56), PARG (PARGi; PDD00017273) (57) and WEE1 (WEE1i; AZD1775; Figure 3A) (58). The time-lapse microscopy proliferation assay was used to screen the OCMs for sensitivity to these four agents as monotherapy (Figure 3B). Since the fallopian tube epithelium is a likely origin for HGSOC, we also included the *hTERT*-immortalized non-ciliated fallopian tube epithelial cell line FNE1 (40,59).

Ten of the 16 OCMs and FNE1 cells were highly resistant to PARGi, meaning GI_50_ could only be calculated for six OCMs (Figure 3B). These results were largely consistent with our prior screen, using colony formation assays (CFA), and collectively we conclude that OCMs 59-3, 72, 109 and 191 are PARGi-sensitive (21). We did however note two exceptions: OCM.195, which displayed sensitivity in the current proliferation analysis, but was resistant by CFA; and OCM.46-6, which was highly sensitive in the current proliferation analysis but only partially sensitive by CFA (21). These exceptions may result from the presence of minor PARGi-resistant clones, which survive treatment to eventually form colonies. While OCM.46-3 was also previously scored as resistant in a proliferation assay, this may reflect the different drug concentration ranges used (21). However, overall consistency with prior studies (15,21), in both measurement of proliferation rate (Figure 1C) and in PARGi-sensitivity (Figure 3B), support use of the proliferation assay in drug-sensitivity profiling the OCMs.

In contrast with PARGi-sensitivity, the panel of OCMs exhibited a more graded response to ATRi, CHK1i and WEE1i, with only the GI_50_ of OCM.109 to ATRi not determined (Figure 3B). FNE1 cells displayed a mid-range GI_50_ to ATRi, CHK1i and WEE1i (Figure 3B, orange bars), indicating that doses required to target the tumour cells may also be detrimental to non-transformed cells. Since HGSOC organoids with replication stress displayed sensitivity to inhibition of either CHK1 or ATR (24), we expected to find a correlation between sensitivity to the four drugs included in our screen. Surprisingly however, for five of the six pairwise comparisons, sensitivity to one drug did not correlate with sensitivity to the other (Pearson's *r* correlation of <0.5; Figure 3C). Therefore, sensitivity is not reflective of a general sensitivity to targeting of the RSR. The one exception was sensitivity to CHK1i and WEE1i (Pearson's *r* correlation of 0.8001), suggesting that there may be a common vulnerability in the OCMs that results in sensitivity to these two agents. Interestingly, GI_50_ to CHK1i also significantly correlated with doubling time (Pearson's r correlation of 0.7941), possibly due to the integral role of CHK1 in cell cycle signalling rendering more rapidly proliferating cells specifically reliant on CHK1 function (Supplementary Figure S3). Nevertheless, because responses generally did not correlate, we conclude that while the RSR may be a frequent vulnerability in HGSOC, drugs targeting the RSR are not simply interchangeable.

### A multiple-low-dose strategy to target the replication stress response

Since monotherapy only targets a small subset of non-overlapping OCMs we decided to evaluate combinations of the RSR inhibitors adopting a multiple-low-dose (MLD) strategy. MLD is an attractive strategy for complete pathway inhibition, whilst potentially minimising emergence of resistance and toxicity to healthy cells (25–27). Indeed, since the GI_50_ of FNE1 cells to ATRi, CHK1i and WEE1i was within the range for the OCMs (Figure 3B), a low-dose combination strategy of these agents may be required clinically to avoid toxicity.

**Figure 4.**
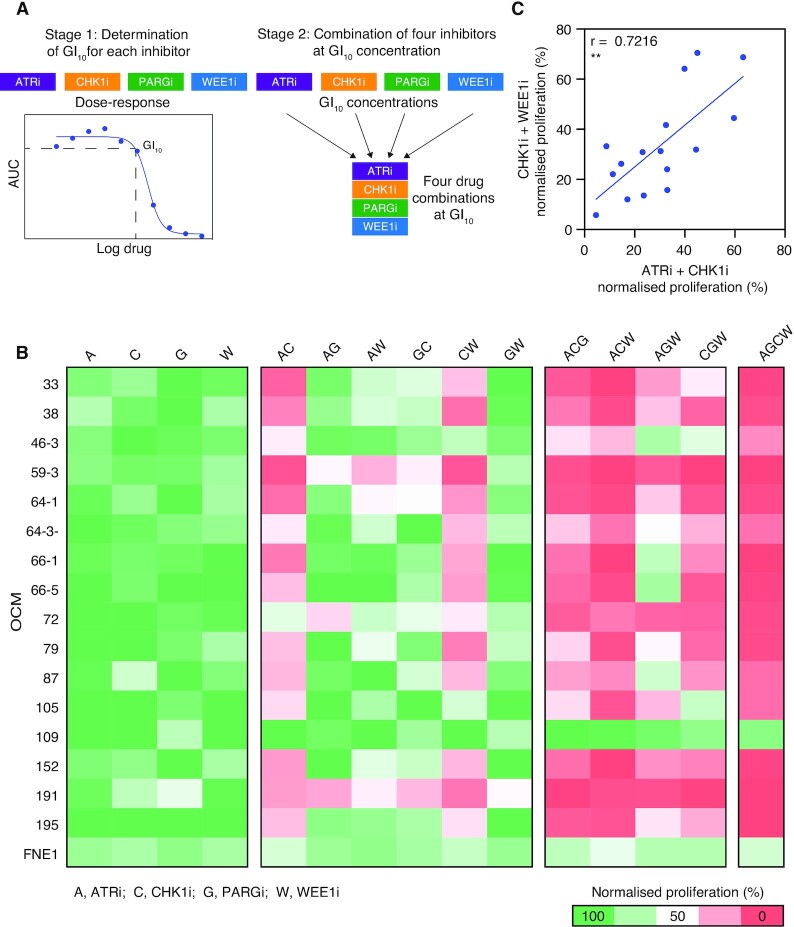
A multiple-low-dose drug combination is anti-proliferative in OCMs. (**A**) The two-stage strategy for the MLD screen. In Stage 1, the time-lapse microscopy drug-sensitivity profiling in Figure 3 was used to determine the GI_10_ dose of the 16 OCMs and FNE1 cell line to PARGi, ATRi, CHK1i and WEE1i. In Stage 2, all possible two-, three- and four-drug combinations were evaluated in the proliferation assay using the GI_10_ doses for each OCM and FNE1 cells defined in Stage 1. (**B**) The 16 OCMs and FNE1 cells were treated with the drug combinations indicated at the GI_10_ doses for 96 h. For each MLD regimen the AUC of the proliferation curve was measured and normalized to that of untreated cells and summarized as % in a heatmap. For OCMs where a GI_10_ could not be determined, a generic low dose was used (i.e. PARGi = 100 nM, ATRi = 2 μM). Data are from three technical replicates. (**C**) Comparison of the normalized AUC of ATRi-CHK1i and CHK1i-WEE1i low-dose combinations for each OCM. Note, OCM.109 is excluded as an GI_10_ dose to ATRi was not calculable. Pearson's r is used to measure correlation. Two-tailed *P* value, ** *P* ≤ 0.01. See also Figure 3 and Supplementary Figure S4.

The MLD screen was conducted in two stages (Figure 4A). Firstly, the 10% maximal growth inhibition dose (GI_10_) of each of the four drugs was determined for each of the 16 OCMs and FNE1 cells, using the dose-response curves generated in the initial screen (Figure 3B). Then, all possible two-, three-, and four-drug combinations were evaluated using the GI_10_ doses. Proliferation was normalised to that of untreated cells and displayed as a heatmap to allow comparison of each MLD regimen (Figure 4B; Supplementary Figure S4A). As expected, GI_10_ doses induced only marginal effects on OCM proliferation. In stark contrast, for 15 of the 16 OCMs, the four-drug MLD combination resulted in a reduction in proliferation of more than 80% versus untreated cells. Thus, while components of the RSR may be redundant, inhibition of the RSR pathway is not tolerated. Two main exceptions include: FNE1 cells, indicating that RSR blockade may be tolerated by non-transformed cells; and OCM.109, suggesting that, in rare cases, some tumour cells can evade RSR blockade. Why OCM.109 is resistant to RSR inhibition remains to be seen (Supplementary Figure S1).

Next, we asked which combinations were driving the effect by calculating the mean reduction in proliferation for each combination across the cohort of OCMs (Supplementary Figure S4B). Of the three-drug combinations, ATRi-PARGi-WEE1i had the smallest impact, suggesting that CHK1i is central in driving the antiproliferative response. Furthermore, since all two-drug combinations containing PARGi had minimal impact, low-dose PARGi is unlikely driving the response. Accordingly, ATRi-CHK1i-WEE1i was the most potent three-drug combination, confirming that CHK1i is required for the strong antiproliferative effect. Indeed, the two-drug combinations of ATRi-CHK1i and WEE1i-CHKi both impart a strong antiproliferative effect on the OCMs. Response to ATRi-CHK1i and WEE1i-CHKi was also strongly correlated (Pearson's *r* of 0.722; Figure 4C), suggesting that sensitivity to both these low-dose combinations may result from the same intrinsic vulnerability. Overall, low-dose combinations of ATRi-CHK1i and WEE1i-CHK1i are sufficient to achieve complete RSR inhibition and have a strong antiproliferative effect in the majority of OCMs.

### Low-dose ATRi-CHK1i is synergistic in OCMs

**Figure 5.**
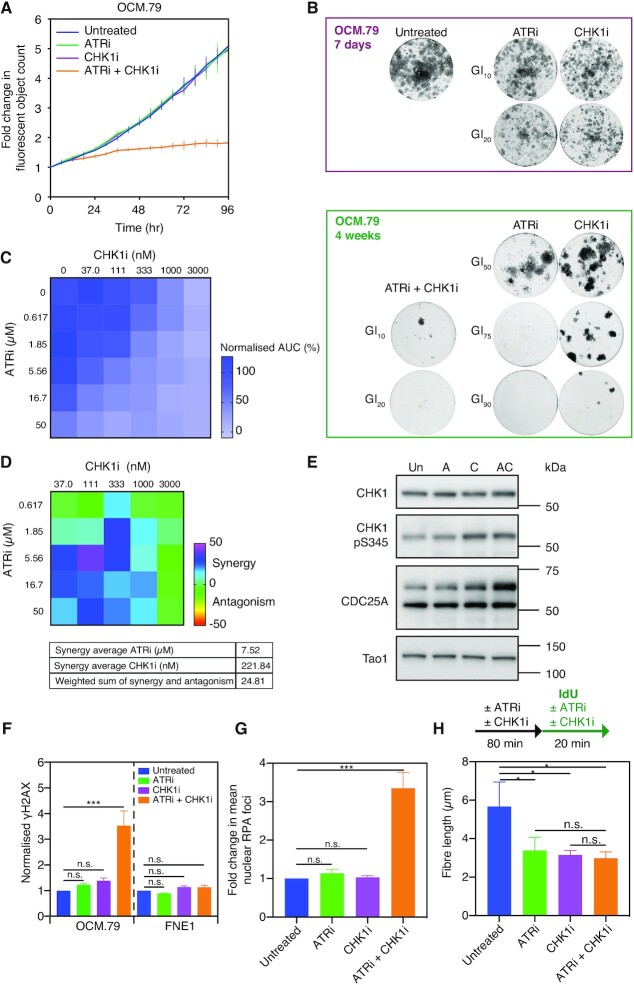
Synergistic low-dose ATRi-CHK1i results in DNA replication stress and catastrophe in patient-derived HGSOC tumour cells. (**A**) Drug-sensitivity profiling assay of OCM.79 using ATRi and CHK1i at GI_10_ concentrations. Fluorescent object count of over 96 hours in the presence and absence of treatment as indicated, normalised to *t* = 0. Data are reproduced from Figure 4B, with each point representing the mean of three technical replicates ± SD. (**B**) Colony formation of OCM.79 following seven days or four weeks treatment with the GI_10_, GI_20_, GI_50_, GI_75_ or GI_90_ doses of ATRi and CHK1i for OCM.79, alone or in combination as indicated. Results are from a single experiment. (**C**) OCM.79 proliferation in a drug-concentration matrix of the indicated doses of ATRi and CHK1i for 72 h using the time-lapse proliferation assay. AUC was normalized to untreated cells. Data are from one technical replicate. (**D**) Matrix of the synergy-antagonism score generated from (C) using the Loewe additivity model. The drug average synergy scores are given to show the localisation of the synergy. The integrated weight sum of synergy and antagonism is also given, representing the total synergy/antagonism measurements across the matrix. (**E**) Immunoblot for CDC25A, CHK1, and CHK1 serine-345 phosphorylation, in OCM.79, with no treatment or after 2h treatment with the GI_10_ doses of ATRi and CHK1i alone or in combination. CDC25A is the upper band of approximately 65 kDa. Tao1 is included as a loading control. Representative immunoblot of two biological replicates. (**F**) Mean nuclear γH2AX intensity by immunofluorescence staining, following 48h treatment of OCM.79 or FNE1 cells with the GI_10_ doses of ATRi and CHK1i alone or in combination, normalized to untreated cells. (**G**) Mean RPA foci per nucleus by immunofluorescence staining, following 48h treatment of OCM.79 with the GI_10_ doses of ATRi and CHK1i alone or in combination, normalized to untreated cells. (**H**) Median length of IdU-labelled nascent DNA fibre in untreated OCM.79, or OCM.79 pre-treated for 80 min with the GI_10_ doses of ATRi and CHK1i alone or in combination (with treatment conditions then continued during 20 min IdU pulse). (F–H) Mean ± SD from three biological replicates, one-way ANOVA, *P ≤ 0.05, ***P ≤ 0.001, n.s. *P* > 0.05. GI_10_, GI_20_, GI_50_, GI_75_ or GI_90_ doses for OCM.79 and FNE1 cells were derived using the proliferation assay. See also Supplementary Figures S5–S7.

The MLD screen indicated that a wide range of distinct OCMs are sensitive to ATRi-CHK1i and CHK1i-WEE1i when combined at GI_10_ doses. This is consistent with previous observations in various cancer models (60–65). Whilst *sequential* low doses of CHK1i and WEE1i has been explored in combination with gemcitabine (66), combined low doses in the absence of chemotherapy are yet to be evaluated clinically. Since the addition of ATR inhibition to CHK1 inhibition is known to induce DNA replication catastrophe in cancer cells (65), we decided to further evaluate the impact of ATRi-CHK1i in the ovarian cancer cells, specifically at low doses. OCM.79 was used as a model, as it had a reduction in proliferation with low-dose ATRi-CHK1i that was representative of the cohort (Figures 4B, 5A). OCM.79 is also from previously treated HGSOC (Stage 3C), in line with most of the cohort, and has wild-type sequence for *BRCA1/2* (with an amplification including *BRCA1* that does not appear to increase expression; Table 1, Supplementary Figure S1) (15).

First, we confirmed the activity of low-dose ATRi-CHK1i in a long-term CFA (Figure 5B). In line with the short-term proliferation assay, GI_10_ or GI_20_ monotherapy had little effect upon colony formation (Figure 5B, upper panel). By contrast, in combination, GI_10_ and GI_20_ had profound impact on colony formation (Figure 5B, lower panel). The low-dose combination proved effective in preventing the long-term appearance of resistance, with only a few colonies present after four weeks treatment with the combined GI_10_ or GI_20_ doses. By contrast, multiple resistant colonies appear following four weeks treatment with the GI_50_ doses of either drug as monotherapy. For CHK1i, resistant colonies also appear after four weeks treatment with the GI_75_ dose. Therefore, CFA confirm the potent activity of low-dose ATRi-CHK1i, and the ability of the low-dose approach to prevent the occurrence of treatment resistance.

To further validate the combination effect, the proliferation assay was used in a drug-concentration matrix with 72 h treatment of OCM.79 (Figure 5C). The synergy matrix, generated using the Loewe Additivity Model (67,68), was dominated by synergy with an overall positive score of 24.8 (Figure 5D). Although more pronounced at higher doses, synergy is predicted for the combined GI_10_ doses of ATRi (2.11 μM) and CHK1i (135.3 nM) for OCM.79. Likewise, a matrix of OCM.33–2 viability was also dominated by synergy (Supplementary Figure S5A, B). We also independently validated synergy by targeting ATR and CHK1 with the alternative inhibitors EPT46464 (69) and LY2603618 (70), respectively. All four possible combinations of ATR and CHK1 inhibitors reduced the viability of OCM.33–2 (Supplementary Figure S5C). Similarly, a synergy matrix of OCM.79 treatment combining increasing doses of ATRi and LY2603618 for 72h resulted in a positive synergy score (Supplementary Figure S5D, E). Although AZD7762 inhibits both CHK1 and CHK2 (56), LY2603618 is a selective CHK1 inhibitor, meaning synergy is the result of CHK1 inhibition (70). Thus, low-dose ATRi-CHK1i is synergistic against ovarian tumours cells in short-term proliferation assays. Taken together with the impact on colony formation, this synergy demonstrates that the ATRi-CHK1i combination induces a potent low-dose combination effect.

### Low-dose ATRi-CHK1i results in DNA replication stress and replication catastrophe

Previous studies have demonstrated that AZD6738 and AZD7762 inhibit ATR and CHK1 activity, respectively (55,56,71). We next confirmed on-target inhibition of ATR and CHK1 in the OCMs, first using OCM.79. Following induction of replication stress with hydroxyurea, ATRi dose-dependently inhibited CHK1 serine-345 phosphorylation by ATR (72) (Supplementary Figure S6A). Similarly, with gemcitabine-induced replication stress, CHK1i dose-dependently inhibited CHK1 serine-296 auto-phosphorylation (73), and destabilisation of CDC25A by CHK1 (74) (Supplementary Figure S6B). Both inhibitors demonstrated activity at dose ranges spanning the GI_10_ doses for OCM.79. In the absence of replication stress induction, the GI_10_ ATRi alone was not sufficient to inhibit basal CHK1 serine-345 phosphorylation (Figure 5E). However, since CHK1 inhibition can abrogate negative feedback on ATR signalling (75), CHK1 serine-345 phosphorylation was increased by GI_10_ CHK1i, or the low-dose combination. Importantly, whilst the stability of CDC25A was unaffected by the ATRi GI_10_ dose, it was stabilised by the CHK1i GI_10_ dose, and possibly further stabilised by the low-dose combination (Figure 5E). Similar results were also obtained in treating OCM.66-1 with the GI_10_ doses (Supplementary Figure S7A).

Having confirmed on target activity, we evaluated whether low-dose ATRi-CHK1i results in DNA damage and subsequent replication stress. Firstly, we stained cells for RPA and γH2AX, markers of single-stranded DNA and double-strand breaks (DSB) respectively, indicating replication stress (76,77). Treatment of OCM.79 with the GI_10_ doses individually resulted in a small, non-significant, increase in γH2AX and RPA staining, compared with untreated cells (Figure 5F, G and Supplementary Figure S6C–F). However, the low-dose ATRi-CHK1i combination caused a significant increase in both markers, with pan-nuclear γH2AX staining indicating prolonged replication stress and subsequent replication catastrophe (76). Likewise, only the low-dose ATRi-CHK1i combination resulted in significant γH2AX staining of OCM.191 (Supplementary Figure S7B, C). Conversely, treatment of FNE1 cells with the GI_10_ doses alone or in combination did not induce γH2AX (Figure 5F).

Since the low-dose combination results in features associated with replication stress, we next evaluated replication fork progression directly using a DNA fibre assay. Following 80 min pre-treatment, cells were pulsed with IdU for 20 min to label active forks and allow measurement of nascent DNA fibres, as an indicator of the speed of replication fork progression (Figure 5H). Compared with untreated cells, fork speed was significantly reduced when OCM.79 was treated with low-dose ATRi or CHK1i alone, or in combination. This slowing of replication fork progression could result from a reduced polymerization rate, or an increase in replication fork stalling. We did examine fork asymmetry, as an indicator of stalling, by labelling origins with BrdU prior to IdU to allow comparison of cognate left and right fork length. However, whilst there was a trend towards increased fork asymmetry with low-dose CHK1i and ATRi-CHK1i, versus no treatment, it was not statistically significant (Supplementary Figure S6H). Nonetheless, the low-dose ATRi-CHK1i combination induces persistent DNA replication stress and eventually replication catastrophe, manifesting as slowed replication forks and pan-nuclear γH2AX, in patient-derived HGSOC tumour cells.

### Low-dose ATRi–CHK1i combination induces post-mitotic cell death

**Figure 6.**
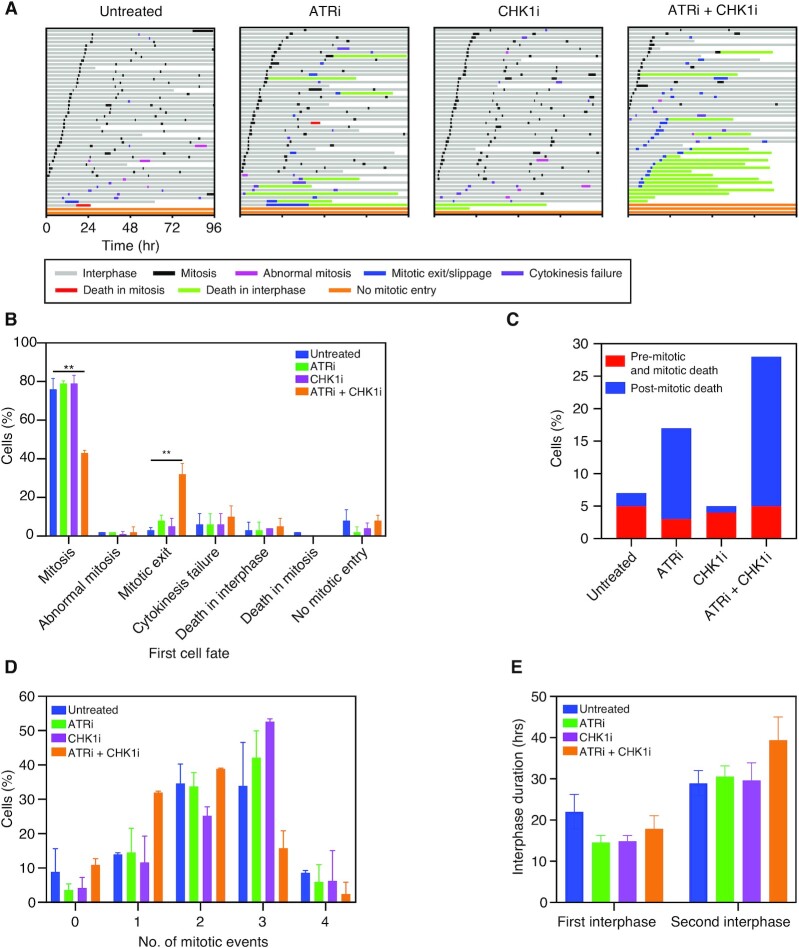
Low-dose ATRi-CHK1i inhibits cell cycle progression and causes post-mitotic cell death. (**A**) Cell fate profiling of OCM.79 using time-lapse microscopy, showing cell behaviour over 96 h with no treatment, or treatment with the GI_10_ doses of ATRi and CHK1i alone or in combination. Horizontal bars each represent a single cell (*n* = 50), with colours indicating cell behaviour. Following mitosis, one daughter cell was randomly selected to continue the analysis. Mitotic exit/slippage occurs when cells enter mitosis but exit without division. Cytokinesis failure occurs when daughters show cleavage furrow regression resulting in merging to one binucleated cell. Other cell division abnormalities such as tripolar cell divisions and division of binuclear cells were recorded as abnormal mitosis. Exemplar profiles shown from one of the two independent experiments described in (B)–(E). (**B**) Summary of first cell fate according to treatment. (**C**) Proportion of cells undergoing pre-mitotic/mitotic cell death (death occurring in the first interphase or mitotic cell death) and post-mitotic cell death (any cell death event that occurred following a normal mitosis, abnormal mitosis, cytokinesis failure and mitotic exit), according to treatment. (**D**) Proportion of surviving cells undergoing one, two, three or four mitotic events according to treatment. A mitotic event was defined as normal or abnormal mitosis, mitotic exit/slippage or cytokinesis failure. (**E**) Duration of the first and second interphase of surviving cells, according to treatment. Note: the first interphase is generally shorter than the second under all conditions as measurement was initiated part way through the first cell cycle. Cells that died during the analysis were excluded from (D) and (E). (B)–(E) are the mean of two independent experiments ± SD, one-way ANOVA, **P ≤ 0.01. Only significant comparisons are indicated, all other comparisons are not significant. GI_10_ doses for OCM.79 were derived using the proliferation assay. See also Supplementary Figure S8.

Inhibition of the replication checkpoint allows replication stress to persist eventually leading to replication catastrophe (76). However, stabilisation of CDC25A suggests that low-dose ATRi-CHKi treatment may also allow cells to bypass the G1/S checkpoint (Figure 5E, Supplementary Figure S7A). Therefore, next we examined cell cycle progression in response to low-dose ATRi-CHK1i in more detail, using time-lapse microscopy over 96 h (Figure 6A) (78). While the majority of OCM.79 cells underwent multiple divisions in the absence of inhibitors, or in the presence of low-dose ATRi or CHK1i alone, only 43% of cells treated with the low-dose combination completed mitosis, with very few cells completing more than one normal division (Figure 6A, B). Rather, 32% of the cells treated with the low-dose combination exited their first mitosis without dividing. Additional anti-proliferative cell fates then followed, with almost all cells that exited mitosis subsequently undergoing a second mitotic exit, cytokinesis failure or death in interphase (Figure 6A). Indeed, post-mitotic death was increased by the low-dose combination, and to a lesser extent by ATRi alone, versus CHK1i alone or no treatment (Figure 6C). Conversely, pre-mitotic and mitotic cell death occurred at a similar rate across all conditions, likely reflecting the intrinsic genomic instability of OCM.79 (15). Thus, post-mitotic cell death is a major contributor to the antiproliferative activity of low-dose ATRi-CHK1i.

To further understand the impact on cell cycle progression, we next focused on cells that survived for the duration of the experiment (Figure 6D, E). Whilst most surviving untreated cells underwent two or three mitotic events during the analysis (including normal or abnormal mitosis, mitotic slippage and cytokinesis failure), there was a shift towards mostly three mitotic events for cells treated with low-dose ATRi or CHK1i alone (Figure 6D). Thus, low doses of the drugs may increase the rate of cell cycle progression. However, most surviving cells treated with the low-dose combination underwent only one or two mitotic events, with only 16% undergoing three events. Subsequent examination of interphase duration found that the first interphase pre-mitosis was shorter in all three treatment groups, versus untreated cells, in line with an initial increase in the rate of cell cycle progression (Figure 6E). Conversely, interphase duration following mitosis was longer with the low-dose combination than with no treatment, but was unaffected by either monotherapy. Therefore, post-mitotic cell cycle arrest likely contributes to the overall reduction in mitotic events with the low-dose combination. Further examination of the distribution of cells according to the duration of second interphase revealed a population of cells with a second interphase of more than 70 h, which may have been exhibiting permanent arrest or senescence (Supplementary Figure S8). Thus, although most analyses shown in 6B–E (derived from two biological replicates) did not reach statistical significance, there is a clear trend suggesting that the anti-proliferative activity of the low-dose combination results from either post-mitotic cell death or induction of post-mitotic cell cycle arrest or senescence.

## DISCUSSION

Acquired resistance to standard-of-care chemotherapy is the major clinical challenge when treating women with HGSOC, and thus there is a pressing need for next-generation, biomarker-led targeted therapies. Due to the paucity of actionable oncogenic driver mutations in this disease, substantial effort is focused on exploiting tumour cell vulnerabilities, in particular the genomic instability that arises from defects in DNA damage repair and RSR pathways. To compare different agents targeting the RSR in the context of HGSOC, we developed and optimized a proliferation-based drug-sensitivity assay to profile a living biobank of patient-derived OCMs. Using this assay to test multiple combinations of four agents targeting different nodes of the RSR, we show that a low-dose two-drug ATRi-CHK1i combination has a broad and potent anti-proliferative effect that warrants further evaluation as a next-generation therapy for HGSOC.

HGSOC displays extensive genomic and phenotypic heterogeneity, features retained by OCMs (15). Despite this heterogeneity, by optimizing several parameters, including seeding density and assay duration, we were able to reproducibly measure OCM proliferation rates. Indeed, the time-lapse imaging-based assay offers several advantages, including the ability to capture cytostatic and cytotoxic effects; high temporal resolution with the ability to interrogate multiple timepoints; and an automated, micro-well format to screen multiple OCMs in parallel in response to multiple drugs. We note however that while fluorescent object count serves as a robust proxy for cell number, there may be confounding issues; for example, bi- or multinucleated cells being counted as distinct entities. To improve the assay, future iterations could explore multiplexing to also measure apoptosis and/or senescence, and use drug-induced growth-rate inhibition to account for differing proliferation rates (49,52,53). Note also that the assay analyses OCMs in exponential growth phase in a well-defined homogeneous 2D cell culture system, which is clearly different from the complex heterogenous 3D microenvironments experienced by tumour cells *in vivo*. Therefore, an important next step will be to adapt the assay to measure proliferation in 3D co-culture systems including stromal cells and matrix components. Nevertheless, because our goal is to exploit intrinsic tumour-cell vulnerabilities, rather than the tumour-microenvironment interactions, this assay represents an important first step in evaluating next-generation therapies.

Screening a panel of OCMs with drugs targeting the RSR components ATR, CHK1, WEE1 and PARG as monotherapies did not reveal any wide-ranging opportunities. While PARGi-sensitivity was bimodal, with six sensitive and 10 highly resistant OCMs, sensitivity to ATRi, CHK1i and WEE1i was graded, with non-transformed FNE1 cells displaying intermediate sensitivity. If RSR inhibitor sensitivity reflects a common replication stress vulnerability, then RSR components may be interchangeable targets (24,76). And indeed, we observed a correlation between sensitivity to WEE1i and CHK1i. Beyond that however, monotherapy sensitivities were not correlated indicating that ATRi, CHK1i, WEE1i and PARGi are not interchangeable, suggesting that RSR inhibitor sensitivity does not reflect a common replication stress vulnerability, but rather independent, context-dependent vulnerabilities particular to a given HGSOC. This poses a tremendous challenge; identifying the cohort of patients likely to benefit from any given RSR inhibitor will require developing a distinct biomarker for each target, rather than an all-purpose RSR signature. Taken together with the observation that only a small subset of OCMs exhibit sensitivity to any given monotherapy, we decided to screen the RSR inhibitors in various combinations on the basis that multi-nodal inhibition might have a broader effect. However, RSR inhibitors have been associated with considerable toxicity in clinical studies, especially in combination with chemotherapy (79). And indeed, as mentioned above FNE1 cells display intermediate sensitivity. Therefore, combinations of RSR inhibitors may yield unacceptable toxicities. To tackle this issue head on, we opted for a MLD approach, with the hypothesis that it would still drive multi-nodal inhibition while also minimizing toxicity as well as the potential for development of resistance. This approach was inspired by two previous low-dose strategies, which showed that targeting four nodes of MAPK pathway in EGFR-mutant lung cancer cells, or combining PI3K, mTOR and MEK inhibitors in clear cell ovarian cancer cells, enabled penetrant pathway inhibition at drug doses that were well-tolerated in animal models (25,26).

This approach was productive: by screening all possible two-, three- and four-drug combinations at their GI_10_ values across a panel of 16 diverse OCMs, we identified two two-drug low-dose combinations, namely ATRi-CHK1i and WEE1i-CHK1i, which potently suppressed proliferation of 15 different OCMs. Importantly, these combinations had minimal effect on non-transformed FNE1 cells. Moreover, OCM.109 was resistant to all the two-, three- and four-drug low-dose combinations, indicating that combination sensitivity is less likely to be a non-specific effect, but rather as result of tumour-cell specific RSR defect. Indeed, sensitivity to low-dose ATRi-CHK1i was accompanied by hallmarks of persistent replication stress, including accumulation of single-stranded DNA, replication fork slowing and DNA damage. Consistent with replication checkpoint override, the first interphase post-drug exposure was accelerated, then often followed by abnormal mitoses, a protracted second interphase, then further abnormal cell divisions or apoptosis. We were intrigued by the rapid mitotic exit of cells treated with the low-dose combination, presumably despite genetic damage that might otherwise be expected to activate the spindle assembly checkpoint (SAC). Whether this reflects a role of CHK1 and/or ATR in the SAC remains to be seen (80–83).

The interaction between ATRi and CHK1i has been demonstrated previously using established cell lines (64,65). In addition, the ATRi-WEE1i combination is synergistic in acute myeloid leukemia, breast, and biliary tract cancer models (84–87). We extend these findings, showing that these two-drug combinations are synergistic in patient-derived models of ovarian cancer, and that they are effective at relatively low concentrations. The observation that ATR activity increases in response to CHK inhibition (Figure 5E, Supplementary Figure S7A) explains why many cancer cells can tolerate CHK1 monotherapy, possibly reflecting the ability of ATR to regulate origin firing, homologous-recombination repair, and/or nucleotide availability (77,88,89). However, in the absence of ATR-dependent compensation, accurate DNA replication is severely compromised. The correlation between ATRi-CHK1i and CHK1i-WEE1i suggests that one or more functions common to ATR and WEE1 can buffer CHK1 inhibition, for example the protection of stalled replication forks via regulation of fork remodeling (90–92). While the mechanism responsible for the synergy between ATRi-CHK1i and CHK1i-WEE1i remains to be fully elucidated, OCM.109 provides an interesting exception to the rule. Future efforts comparing multi-omic and cell biology parameters in OCM.109 with models that are sensitive to the two-drug combination could be informative. In the meantime, it is encouraging that 15 out of 16 OCMs are sensitive to the two two-drug combinations, meaning that these approaches may have broad reach therapeutically. Moreover, if the vast majority of HGSOC are sensitive to MLD RSR inhibition due to the high prevalence of replication stress (23,24), the search for a relevant biomarker may be simplified or even bypassed. Note that sensitive OCMs included ones that were both *BRCA*-mutant and wildtype (Supplementary Figure S1); as such these low-dose combinations may be an option for HGSOC that are intrinsically PARPi resistant or those that acquire PARPi resistance. Another advantage of the low-dose approach is that multi-node inhibition may block evolutionary escape routes, thus minimizing the potential for drug-resistant subclones to emerge (25), a notion supported by the marked reduction in long-term colonies forming in the low-dose ATRi-CHK1i combination, compared with the high-dose monotherapies (Figure 5B). Whether the low dose approach alleviates toxicity remains to be seen although we note that an ATRi-CHK1i combination was well tolerated in a lung cancer xenograft model (65). An important next step will be to examine low-dose ATRi-CHK1i *in vivo*; importantly, several of the OCMs described here do engraft in mice (unpublished data), so testing this concept *in vivo* will be possible in the future.

## DATA AVAILABILITY

For Supplementary Figure S1, previously published RNA sequencing from the 16 OCMs is available from EBML-EBI using accession numbers E-MTAB-7223, E-MTAB-10801, and E-MTAB-11000 (FASTQ files available from European Nucleotide Archive under Study Accessions PRJEB28709, PRJEB47842, PRJEB46736) (15,20,21). Published exome sequencing from 11/16 OCMs is available from EBML-EBI using accession number E-MTAB-7225 (FASTQ files available from European Nucleotide Archive under Study Accession PRJEB8710) (15).

## Supplementary Material

zcac036_Supplemental_File
