# Supplementary material for: Multiple-low-dose therapy: effective killing of high-grade serous ovarian cancer cells with ATR and CHK1 inhibitors
Source: NAR Cancer. 2022 Nov 12;4(4):zcac036. doi: 10.1093/narcan/zcac036 (PMC9653014; doi:10.1093/narcan/zcac036)
Supplement: zcac036_Supplemental_File [file zcac036_supplemental_file.pdf]

A

Patient	Disease	OCM	Treatment	Immunofluorescence			Immunoblot			*TP53* Sanger (RT-PCR)	FACS				GI_{50} (Mean)	
				PAX8	p53 ± Nutlin-3		p53 ± Nutlin-3		Myc		CA125	Epcam	CD44	CD105	Cisplatin (μM)	Paclitaxel (nM)
					-	+	-	+								
33[1]	HGSOC	33-2	Y							Inframe insertion					0.3	5.8
38[1]	HGSOC	38	N							Inframe deletion					0.4	8.3
46[1]	HGSOC	46-3	Y							Frameshift					1.1	6.2
59[1]	HGSOC	59-3	Y							Frameshift					2.1	4.0
64[1]	Possible HGSOC/ LGSOC mix[3]	64-1	Y							p.V216M					1.5	14.0
		64-3-	Y												0.6	5.5
66[1]	HGSOC	66-1	Y							p.Y163C					1.3	8.7
		66-5	Y												1.1	7.3
72[1]	MUC	72	Y							p.D281E					1.2	6.4
79[1]	HGSOC	79	Y							Deletion					1.4	7.8
87[1]	Possible CCOC[3]	87	N							Wildtype					0.5	7.2
105[2]	HGSOC	105	Y							p.Q105P					1.3	4.4
109[2,3]	HGSOC	109	Y							p.R248Q					1.1	6.9
152[2,3]	Serous adenocarcinoma[3]	152	Y							p.Y220C					1.2	3.3
191[2,3]	HGSOC	191	Y							p.R248Q					0.7	5.8
195[2,3]	Possible LGSOC[3]	195	N	W						Wildtype						

B

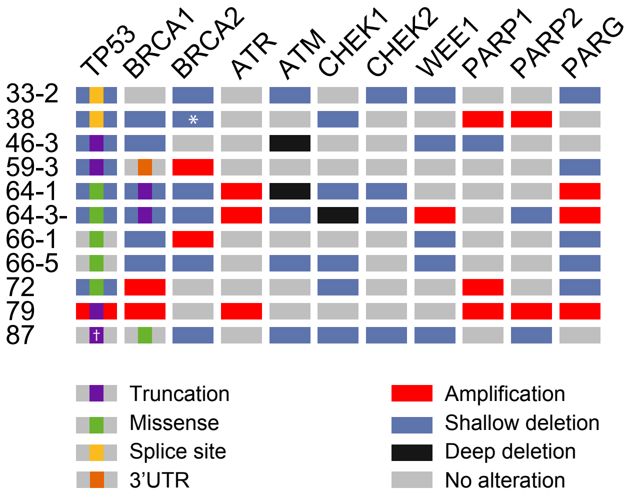

C

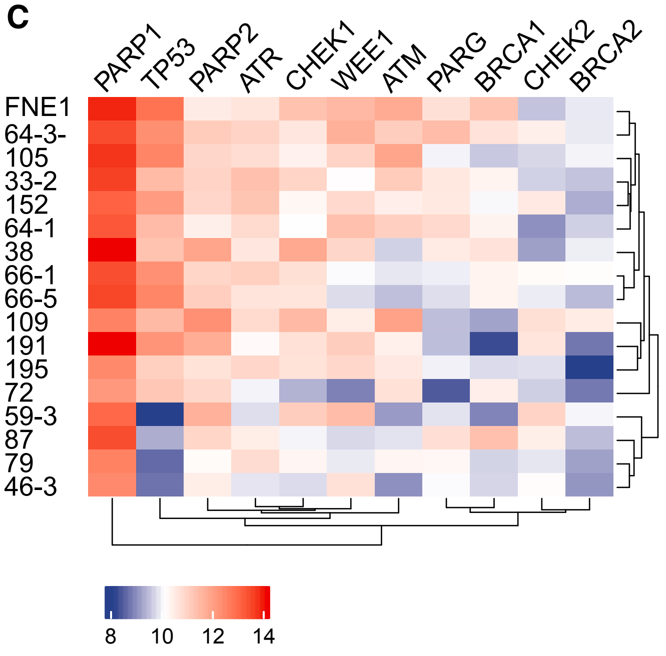

Supplementary Figure 1

Figure S1: Characteristics of OCMs

(**A**) Table summarising OCM characteristics from (1) Nelson *et al.*, 2020, (2) Coulson-Gilmer *et al.*, 2021; (3) Barnes *et al.*, 2021. Note that the culture established from the third biopsy from patient 64 was further separated into EpCAM-negative (64-3-) and EpCAM-positive cells (64-3+), with OCM.64-3- included in the current analysis. Disease: HGSOC, high-grade serous ovarian cancer; CCOC, clear cell ovarian cancer; MUC, mucinous ovarian cancer. Treatment: N, chemo-naïve. Immunofluorescence for PAX8 and p53 (with and without the Mdm2 inhibitor Nutlin-3): blue=present; white=absent and dark blue indicates functional p53 induction by Nutlin-3 (no change with Nutlin-3 indicates lack of functional p53 induction). Immunoblot for p53±Nutlin-3 uses the same colours as for IF; Immunoblot for Myc: strong=dark blue; medium=intermediate blue; weak=light blue; or absent=white. *TP53* Sanger: *TP53* mutations identified by Sanger sequencing of RT-PCR products. FACS: flow cytometry quantitating the number of cells positive for the markers indicated, where white indicates <5% and increasingly darker shades of red indicate 5–25, 25–50, 50–75, >75% of cells. GI_{50} for cisplatin and paclitaxel are mean of at least 3 biological replicates using the proliferation assay. W, weak. (**B**) Single nucleotide variants, small insertions and deletions, and copy number variation as visualised by oncoprint for the 11 OCMs with exome sequencing available (Nelson *et al.*, 2020). *We previously described OCM.38 as having a *BRCA2* mutation, Nelson *et al.*, 2020, which was not apparent when we reanalysed the exome sequencing data here, possibly due to the use of different threshold parameters (note however a *BRCA2* mutation is detected in the RNA sequencing data); †We previously found OCM.87 to have wildtype *TP53*, Nelson *et al.*, 2020, however a truncation mutation is detected at low frequency (33%) under the current parameters, suggesting some heterogeneity. (**C**) Heatmap showing expression levels of 11 genes in the OCMs using RNA sequencing from Nelson *et al.*, 2020; Coulson-Gilmer *et al.*, 2021; Barnes *et al.*, 2021. High or low expression is indicated by red or blue colouration, respectively. Key shows variance stabilising transformed.

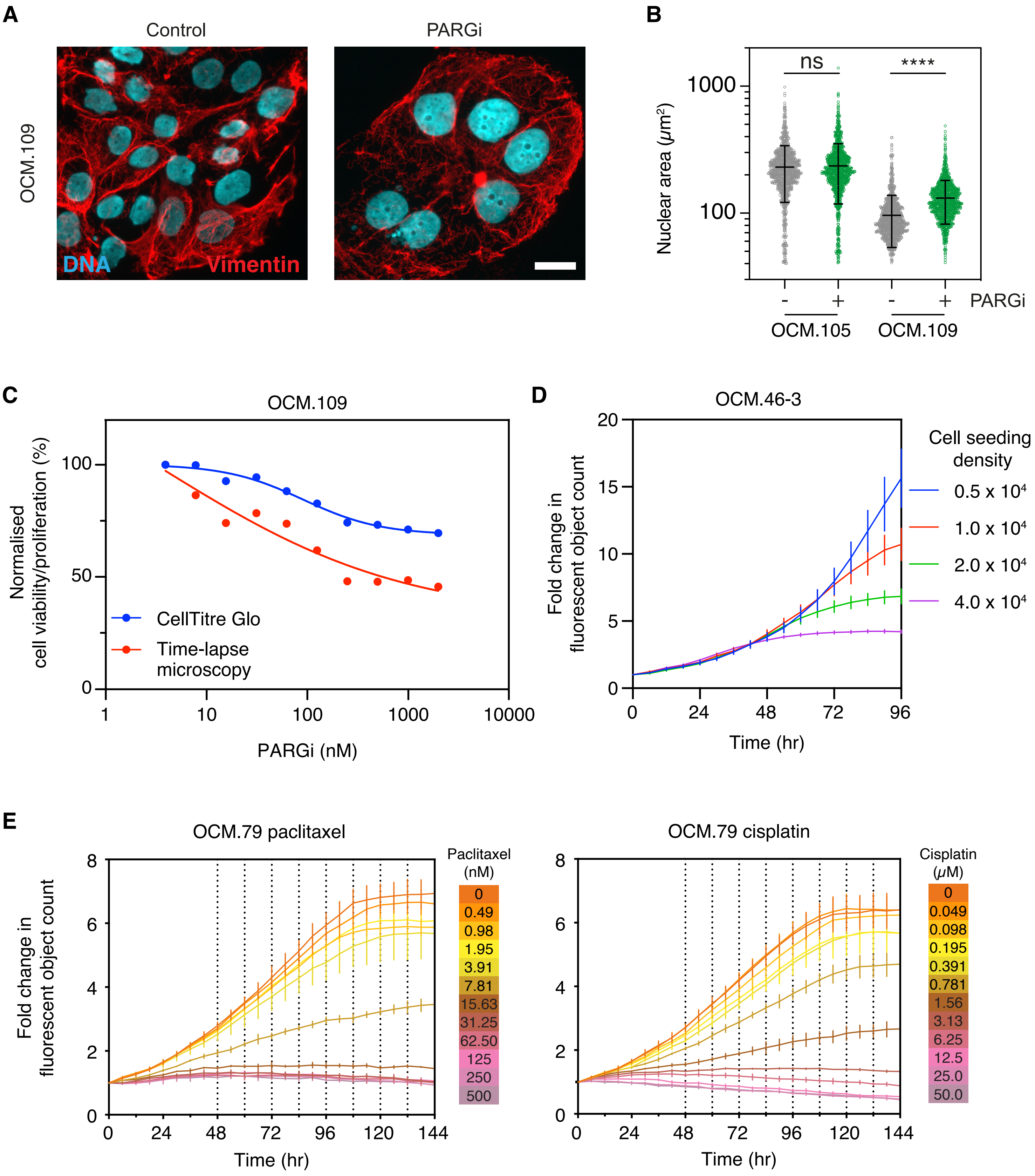

Supplementary Figure 2

Figure S2: Drug sensitivity profiling of OCMs by time-lapse microscopy

(**A**) Immunofluorescence of PARGi-sensitive OCM.109 following 72h treatment with DMSO (Control) or 1 µM PARGi (PDD00017273). Fried egg morphology with treatment evidenced by nuclear size. Merged DNA and vimentin fluorescence images are shown. Scale bar 20 µm. (**B**) Quantification of nuclear area following 72h treatment of PARGi-resistant OCM.105 and PARGi-sensitive OCM.109 with 1 µM PARGi or DMSO. Hoechst 33258 staining was used to calculate intensity thresholds and generate a nuclear mask from which nuclear area could be calculated. One thousand nuclei were analyzed from one biological replicate. Lines represent the mean ± SD. One-way ANOVA, ****$p \leq 0.0001$, n.s. $p > 0.05$. (**C**) Comparison of metabolism-based CellTiter-Glo®, and time-lapse microscopy-based, PARGi-sensitivity profiling of OCM.109. CellTiter-Glo® and time-lapse microscopy assays measure cell viability and proliferation, respectively, with both metrics normalised to untreated cells. (**D**) Proliferation of untreated OCM.46-3 over 96h, normalised to t=0, at four cell seeding densities. Each point represents the mean of six technical replicates ± SD. (**E**) Proliferation of OCM.79 over 144 hours in response to paclitaxel and cisplatin, normalised to t=0. Dotted vertical lines indicate the 12-hour intervals at which GI_{50} was determined. Each point represents the mean of three technical replicates ± SD. See also Figure 2.

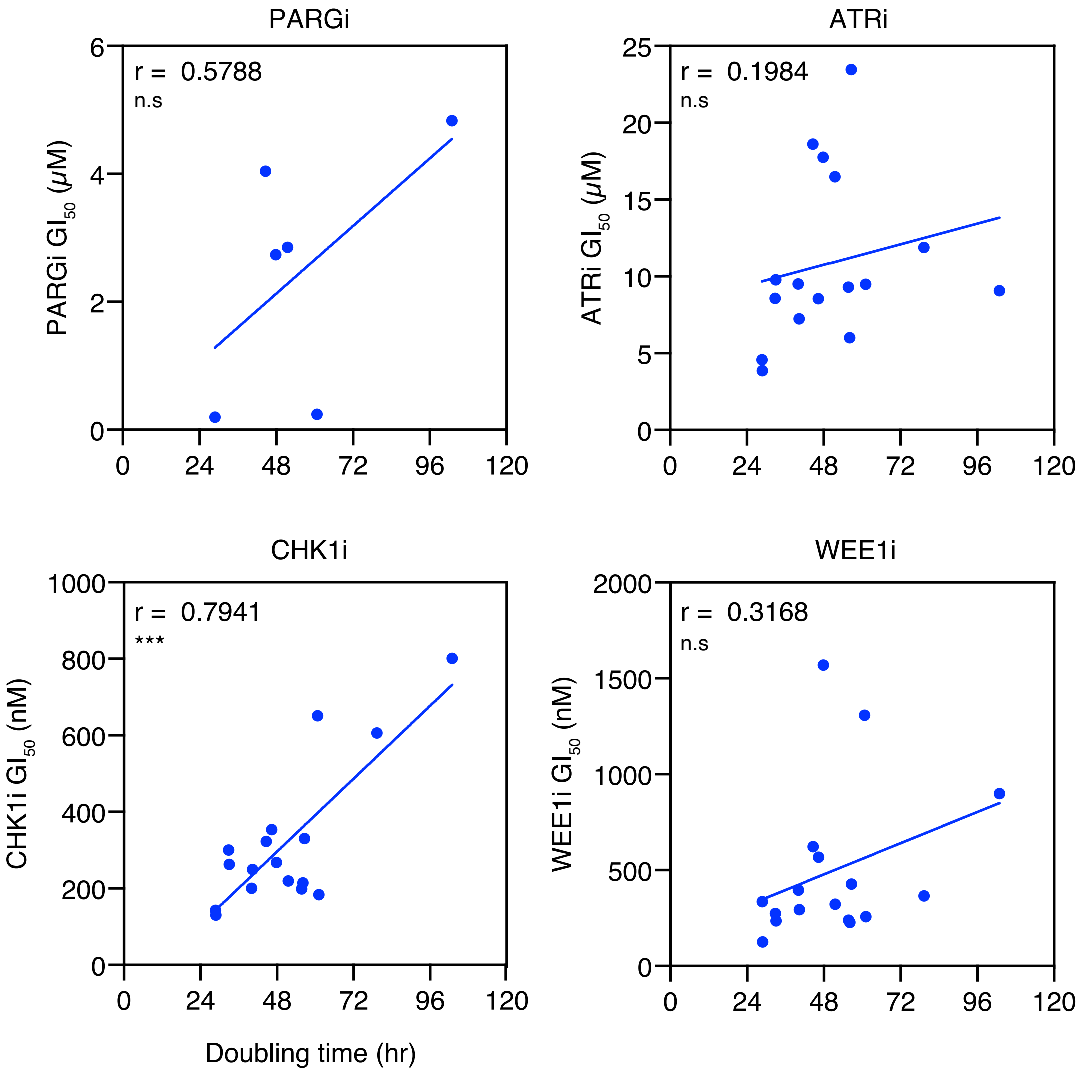

Supplementary Figure 3

Figure S3: Comparison of OCM doubling time and GI_{50} to PARGi, ATRi, CHK1i, and WEE1i

Comparison of OCM doubling time from Figure 1B with OCM GI_{50} for PARGi, ATRi, CHK1i and WEE1i calculated from time-lapse drug-sensitivity profiling in Figure 3B. Pearson's r is used to measure correlation. Two-tailed P value, *** $p \leq 0.001$, n.s. $p > 0.05$. See also Figures 1 and 3.

A

Normalised proliferation (%) of the OCMs in the MLD screen

OCM	A	C	G	W	AC	AG	AW	GC	CW	GW	ACG	ACW	AGW	CGW	AGCW
33-2	89	82	102	95	9	93	66	61	33	104	7	0	23	45	1
38	74	93	111	77	17	84	63	69	12	98	14	2	33	9	3
46-3	88	102	97	92	45	95	93	82	71	91	42	31	77	61	19
59-3	87	94	93	79	5	48	29	45	6	73	4	0	7	0	0
64-1	97	83	102	78	11	88	48	51	22	88	5	2	35	5	3
64-3	116	96	89	83	44	98	66	107	32	72	35	13	52	29	13
66-1	97	92	96	102	15	94	97	82	26	111	13	0	71	19	0
66-5	128	92	102	109	33	101	103	76	24	99	10	3	79	6	1
72	109	133	95	99	60	39	68	58	45	74	8	14	9	8	2
79	102	100	92	77	33	100	57	90	16	69	39	3	48	11	3
87	99	66	109	89	30	94	109	64	31	89	24	19	66	21	11
105	113	115	91	107	40	101	77	100	64	102	40	5	31	68	12
109	112	118	72	103	119	95	110	80	102	73	107	99	94	84	87
152	90	85	108	78	23	102	60	68	31	106	12	0	20	17	1
191	96	71	58	100	24	26	45	31	14	49	1	4	4	1	0
195	111	117	105	115	33	86	83	77	42	101	7	5	43	28	0
FNE1	82	77	86	78	63	81	84	78	69	82	71	57	74	74	65

B

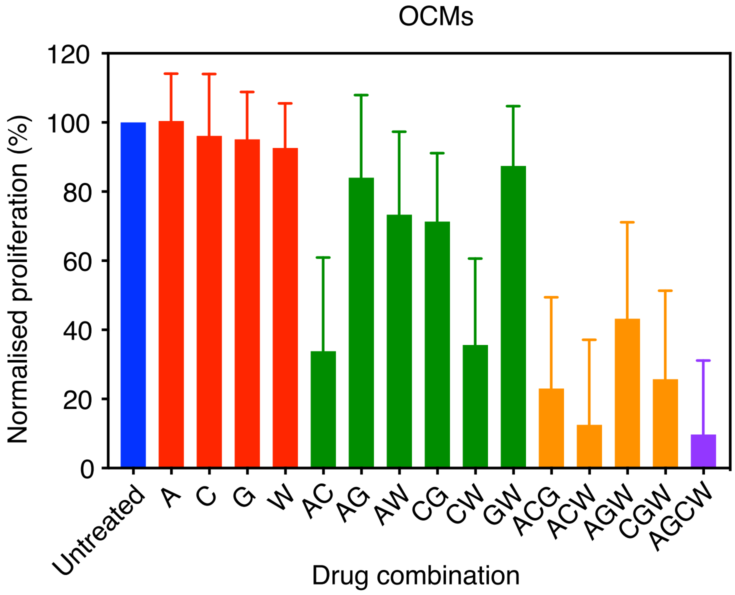

C

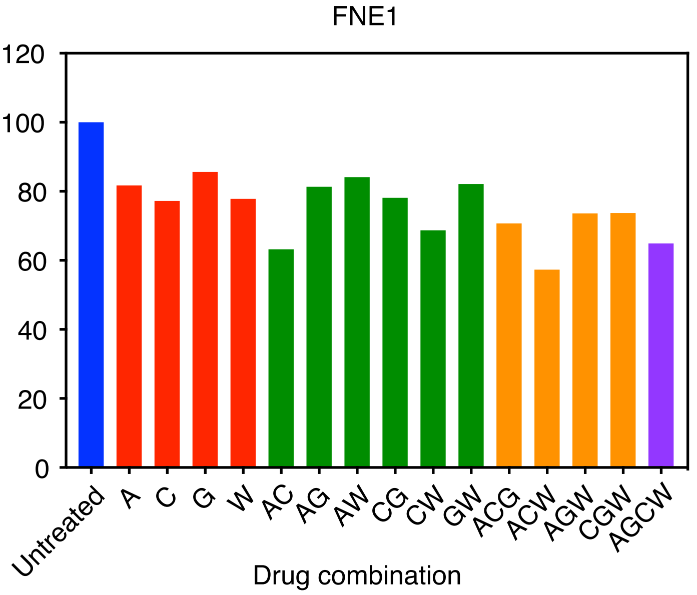

A, ATRi; C, CHK1i; G, PARGi; W, WEE1i

Supplementary Figure 4

Figure S4: Mean drug-sensitivity across OCMs demonstrates activity of low-dose ATRi-CHK1i and WEE1i-CHKi combinations

(**A**) Normalised proliferation (%) of the OCMs and FNE1 cells in the MLD screen. The 16 OCMs and FNE1 cells were treated with the drug combinations indicated at the GI_{10} doses for 96 hours. The time-lapse microscopy drug-sensitivity profiling in Figure 3 was used to determine the GI_{10} doses. For each MLD regimen the AUC of the proliferation curve was measured and normalised to that of untreated cells. For OCMs where a GI_{10} could not be determined, a generic low dose was used (i.e. PARGi = 100 nM, ATRi = 2 μM). These data are presented as a heatmap in Fig.4B. (**B**) The proliferation of individual OCMs for each MLD regimen was normalized to the corresponding untreated condition, then a mean % across all OCMs calculated. Mean ± SD is shown. (**C**) Reduction in proliferation of FNE1 cells, compared with untreated FNE1 cells, for each MLD regimen (reproduced from Fig.4B and S4A). All data are from three technical replicates. A, ATRi; C, CHK1i; G, PARGi; W, WEE1i. See also Figure 4.

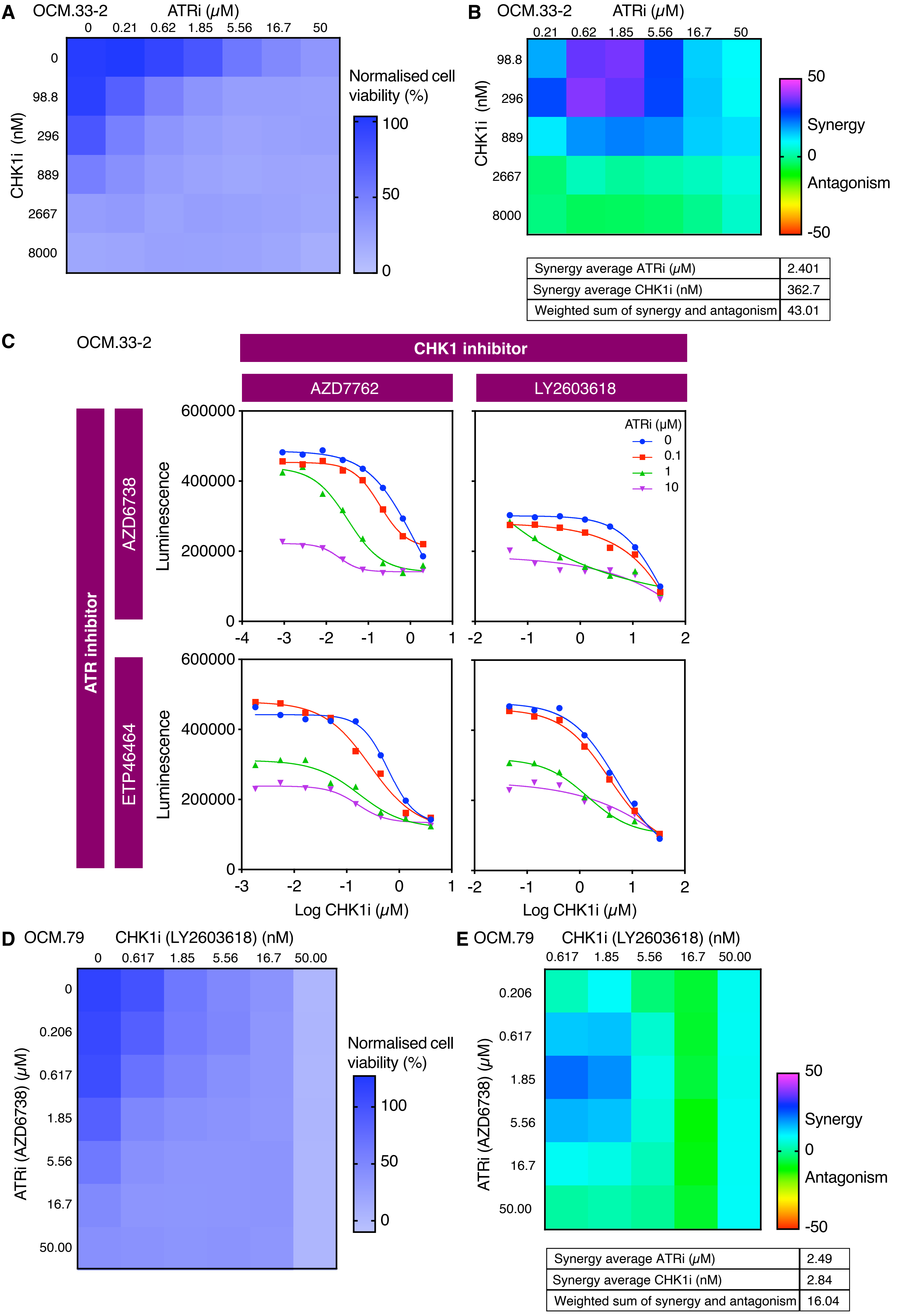

Supplementary Figure 5

Figure S5: **Validation of ATRi-CHK1i using alternative OCMs and inhibitors**

(**A**) OCM.33-2 viability in a drug concentration matrix of the indicated doses of ATRi and CHK1i for 72h using the cell viability assay. Luminescence was normalized to untreated cells. Data are from two technical replicates. (**B**) Matrix of the synergy-antagonism score generated from (**A**) using the Loewe additivity model. (**C**) Viability (luminescence) of OCM.33-2 under a titration of the CHK1 inhibitors AZD7762 or LY2603618 with the addition of ATR inhibitors AZD6738 and ETP46464 at the indicated doses. Viability was measured after 72h using the cell viability assay, normalised to untreated cells. Note,10 µM of either ATR inhibitor induced a reduction in viability in the absence of CHK1 inhibition. Data are from one technical replicate. (**D**) OCM.79 viability in a drug-concentration matrix of the indicated doses of ATRi and the alternative CHK1 inhibitor, LY2603618, for 72h using the cell viability assay. Luminescence was normalized to untreated cells. Data are from one technical replicate. (**E**) Matrix of the synergy-antagonism score generated from (**D**) using the Loewe additivity model. For (**B**) and (**E**) the drug average synergy scores are given to show the localisation of the synergy. The integrated weight sum of synergy and antagonism is also given, representing the total synergy/antagonism measurements across the matrix. Note: while the sum of synergy and antagonism score for the AZD6738/LY2603618 combination is lower than for AZD6738/AZD7762 in Figure 5, the proliferation metrics were acquired by different methods (cell viability assay and time-lapse microscopy assay respectively), therefore caution must be taken when comparing results directly. See also Figure 5.

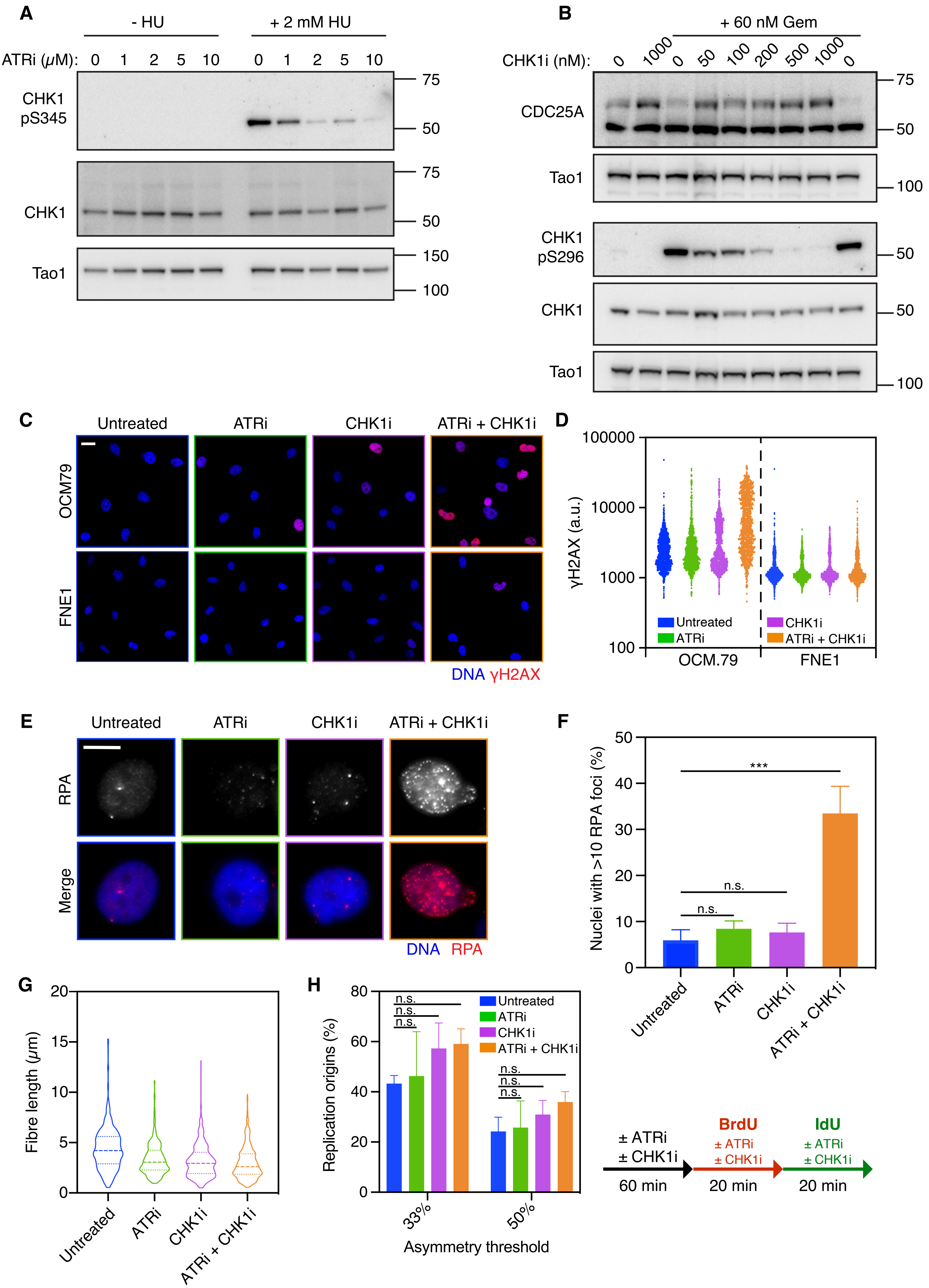

Supplementary Figure 6

Figure S6: **Low-dose ATRi-CHK1i results in DNA replication stress and catastrophe in patient-derived HGSOC tumour cells**

(**A**) Immunoblot showing dose-dependent inhibition of CHK1 serine-345 phosphorylation in OCM.79 after treatment for 1h with ATRi, then 1h with ATRi ± 2 mM hydroxyurea (HU). Concentrations of ATRi used are shown. (**B**) Immunoblot showing dose-dependent inhibition of CHK1 serine-296 phosphorylation and CDC25A destabilization in OCM.79 after treatment for 24h with 60 nM gemcitabine, then 1h with CHK1i ± 60 nM gemcitabine. Concentrations of CHK1i used are shown. CDC25A is the upper band of approximately 65 kD. (A) and (B) show representative immunoblots of two biological replicates, with total CHK1 and Tao1 included as loading controls. (**C**) Representative immunofluorescence images of γH2AX foci in OCM.79 (upper panels) in response to no treatment or 48h treatment with the GI_{10} doses of ATRi and CHK1i alone, and pan-nuclear staining resulting from the low-dose combination. Lower panels show identical treatment of FNE1. Scale bar 20 µm. (**D**) Quantification of mean nuclear γH2AX intensity following no treatment or 48h treatment of OCM.79 and FNE1 cells, with the GI_{10} doses of ATRi and CHK1i alone or in combination. One thousand nuclei were analyzed from one biological replicate. (**E**) Representative immunofluorescence images of RPA1 (RPA70) foci in OCM.79 in response to no treatment or 48h treatment with the GI_{10} doses of ATRi and CHK1i alone and in combination. Scale bar 10 µm. (**F**) Quantification of cells with >10 RPA1 (RPA70) foci per nucleus in OCM.79 in response to no treatment or 48h treatment with the GI_{10} doses of ATRi and CHK1i alone or in combination. Mean ± SD from three biological replicates. (**G**) Distribution of IdU-labelled nascent DNA fibre length in untreated OCM.79 or OCM.79 pre-treated for 80 min with the GI_{10} doses of ATRi and CHK1i alone or in combination (with treatment conditions then continued during 20 min IdU pulse), from one biological replicate. Lines denote the median and lower and upper quartiles. (**H**) Percentage of replication origins showing sister replication fork asymmetry, in untreated OCM.79, or OCM.79 pre-treated for 1h with the GI_{10} doses of ATRi and CHK1i, alone or in combination. Treatment conditions were then continued during sequential pulses of BrdU and IdU (20 min each). Fork asymmetry is defined as a fold change between cognate left and right nascent IdU-labelled fork length of either >33% or >50%. Bars represent the mean average of three biological replicates ± S.D. For G and H, a minimum of 300 fibres were analyzed for each condition. For F and H, one-way ANOVA, ***$p \leq 0.001$, n.s. $p > 0.05$. GI_{10} doses for OCM.79 and FNE1 cells were derived using the proliferation assay. See also Figure 5.

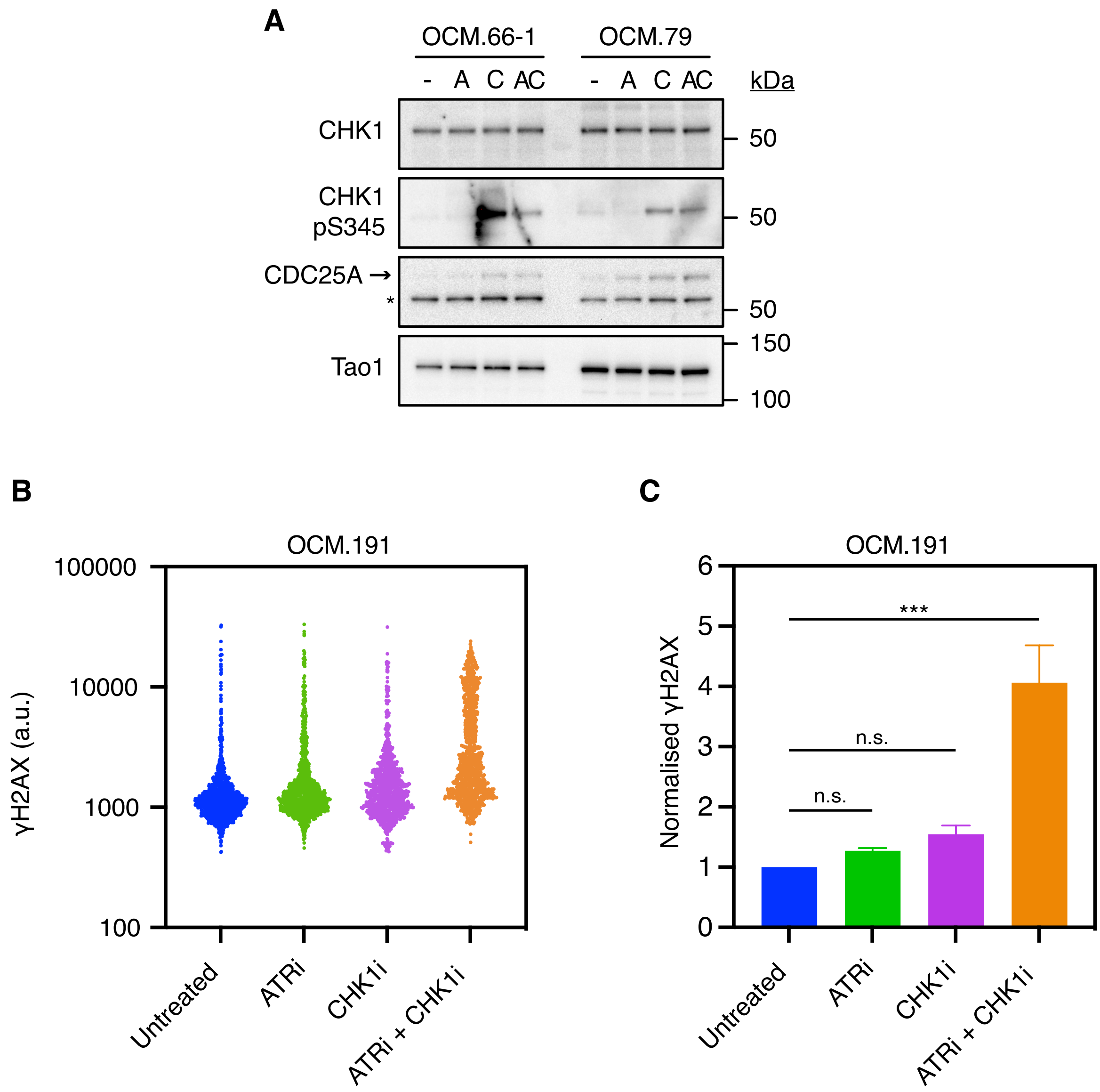

Supplementary Figure 7

Figure S7: **Validation of ATRi-CHK1i-induced replication catastrophe in OCMs**

(**A**) Immunoblot for CDC25A, CHK1, and CHK1 serine-345 phosphorylation in OCM.66-1 and OCM.79, with no treatment or after 2h treatment with the GI_{10} doses of ATRi and CHK1i alone or in combination. CDC25A is the upper band of approximately 65 kD, with (*) indicating a non-specific band. Tao1 is included as a loading control. Representative immunoblot of one biological replicate. (**B**) Quantification of mean nuclear γH2AX intensity following no treatment or 48h treatment of OCM.191 with the GI_{10} doses of ATRi and CHK1i alone or in combination. One thousand nuclei were analyzed from one biological replicate. (**C**) Mean nuclear γH2AX intensity by immunofluorescence staining, following 48h treatment of OCM.191 cells with the GI_{10} doses of ATRi and CHK1i alone or in combination, normalized to untreated cells. Mean ± SD from three biological replicates, one-way ANOVA, ***$p \leq 0.001$, n.s. $p > 0.05$. GI_{10} doses were derived using the proliferation assay. See also Figure 5.

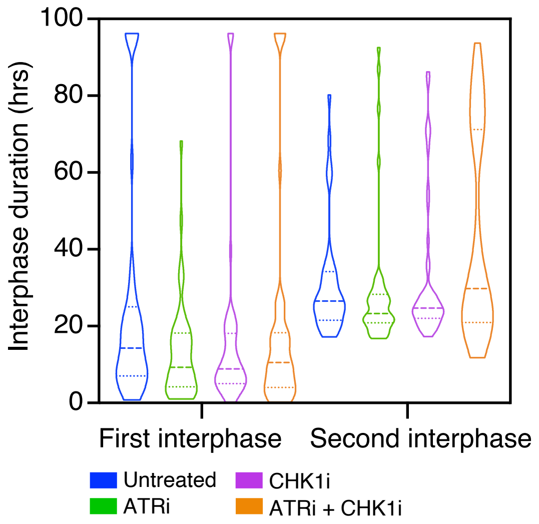

Supplementary Figure 8

Figure S8: **The low-dose ATRi-CHK1i combination inhibits cell cycle progression**

Violin plot displaying the distribution of interphase duration in individual cells, according to treatment, comparing the first interphase and second interphase from one independent cell fate profiling experiment, as described in Figure 6A. The first interphase included no mitotic entry events, with all interphases that ended in death in interphase excluded from the analysis. Lines denote the median, lower and upper quartiles. A minimum of 38 interphases were analysed for each condition. OCM.79 was treated with GI_{10} doses of ATRi and CHK1i, alone or in combination, derived using the proliferation assay. See also Figure 6.
